# TAT-HSP27 Peptide Improves Neurologic Deficits and Reduces Apoptosis After Experimental Subarachnoid Hemorrhage

**DOI:** 10.3389/fncel.2022.878673

**Published:** 2022-04-28

**Authors:** Xiao-yan Zhou, Jing-yi Sun, Wei-qi Wang, Shu-xian Li, Han-xia Li, Hui-juan Yang, Ming-feng Yang, Hui Yuan, Zong-yong Zhang, Bao-liang Sun, Jin-Xiang Han

**Affiliations:** ^1^Department of Biochemistry and Molecular Biology, School of Basic Medical Sciences, Shandong University, Ji'nan, China; ^2^Department of Neurosurgery, First Affiliated Hospital of Shandong First Medical University and Shandong Academy of Medical Sciences, Ji'nan, China; ^3^Biomedical Sciences College and Shandong Medicinal Biotechnology Centre, Shandong First Medical University and Shandong Academy of Medical Sciences, Ji'nan, China; ^4^Key Lab for Biotech-Drugs of National Health Commission, Shandong First Medical University and Shandong Academy of Medical Sciences, Ji'nan, China; ^5^Department of Orthopedics, Shandong Provincial Hospital Affiliated to Shandong First Medical University and Shandong Academy of Medical Sciences, Jinan, China; ^6^Department of Neurology, Shandong Provincial Hospital Affiliated to Shandong First Medical University and Shandong Academy of Medical Sciences, Jinan, China; ^7^Department of Neurology, Key Laboratory of Cerebral Microcirculation, Second Affiliated Hospital of Shandong First Medical University and Shandong Academy of Medical Sciences, Taian, China

**Keywords:** subarachnoid hemorrhage, HSP27, cell apoptosis, neurologic deficits, TAT-HSP27_65−90_ peptide

## Abstract

Cell apoptosis plays an important role in early brain injury (EBI) after subarachnoid hemorrhage (SAH). Heat shock protein 27 (HSP27), a member of the small heat shock protein family, is induced by various stress factors, and has a protective effect on cells. However, the role of HSP27 in brain injury after SAH needs to be further clarified. Here, we report that HSP27 level of cerebrospinal fluid (CSF) is clearly increased at day 1 in patients with aneurysmal SAH (aSAH). That increase is related to the clinical severity of the damage, as assessed by the grade of Hunt and Hess (HH), World Federation of Neurological Surgeons (WFNS) and Fisher. In the rat SAH model, HSP27 of CSF is increased at first and then declined; overexpression of *Hsp27*, not knockdown of *Hsp27*, attenuates SAH-induced neurological deficit and cell apoptosis in basal cortex; overexpression of *Hsp27* effectively suppresses SAH-elevated the activation of Mitogen-Activated Protein Kinase Kinase 4 (MKK4), the c-Jun N-terminal kinase (JNK), c-Jun and caspase-3. In an *in vitro* hemolysate-damaged cortical neuron model, HSP27_65 − 90_ peptide effectively inhibits hemolysate-induced neuron death. Furthermore, TAT-HSP27_65 − 90_ peptide, a fusion peptide consisting of Trans-Activator of Transcription (TAT) of HIV and HSP27_65 − 90_ peptide, effectively attenuates SAH-induced neurological deficit and cell apoptosis in basal cortex in rats. Altogether, our results suggest TAT-HSP27 peptide improves neurologic deficits and reduces apoptosis.

## Introduction

Subarachnoid hemorrhage (SAH) is a subtype of stroke with high mortality and morbidity rate, which is mainly caused by the rupture of intracranial aneurysms (Chen et al., 2014). Although ruptured aneurysms are treated by clipping or coiling surgically, 67% of patients with SAH still have neurological sequelae due to early brain injury (EBI) and delayed brain injury (Sehba et al., 2012; Fujii et al., 2013). Cell apoptosis is the most important pathophysiological change underlying EBI, which initiates complex signaling pathways that lead to neuronal death (Sehba et al., 2012; Fujii et al., 2013; Zhang et al., 2015). Therefore, identifying critical pro-death signaling cascades and finding neuroprotective agents targeting this cascade have become an important strategy to against EBI after SAH.

Heat shock proteins are evolutionarily conserved molecular chaperones that consists HSP 40, HSP60, HSP70, HSP90, and small HSPs, which have critical role in stress response (Shan et al., 2020). HSP27 is a member of the small HSP family, has molecular chaperone activity (Kostenko and Moens, 2009), decreases protein aggregation and helps degradation by the proteasome, suppresses release of cytochrome c and caspase activation (Shan et al., 2021), and also exerts cytoprotective effect through cytoskeleton stabilization and antioxidant activity (Vendredy et al., 2020). Previous studies demonstrated that overexpression of HSP27 provides neuroprotection in multiple neurological disease models, include cerebral ischemia (An et al., 2008; Stetler et al., 2008; Shi et al., 2017), kainate-induced neuronal death, and Alzheimer's disease (Akbar et al., 2003; Toth et al., 2013), which were mainly credited to its anti-apoptotic effect. Under SAH pathology, the change of expression and phosphorylation of HSP27 in brainstem and cerebral vessels has been observed in the SAH model (Macomson et al., 2002; Satoh et al., 2003), suggesting that HSP27 is associated with brain injury. However, the potential neuroprotective role of HSP27 has not been illustrated in SAH.

In this study, we measured the concentration of HSP27 in cerebrospinal fluid (CSF) from patients with aneurysmal SAH (aSAH) and then assessed the expression of HSP27 and investigated the effect of knockdown or overexpression of HSP27 on neurological deficit in rat SAH model. We investigated the effect of small peptides from HSP27 on cell apoptosis in an *in vitro* hemolysate-damaged cortical neuron model. We next explored the effect of TAT-HSP27_65−90_ peptide on neurological deficit and cell apoptosis in rat SAH model.

## Materials and Methods

### Patients and CSF Collection

After approval by the ethical committee of Shandong Provincial Hospital, an observational study of CSF from 73 patients with SAH (confirmed by a head computed tomography angiography) between May 2019 and May 2020 was performed. Inclusion criteria: (1) aneurysm treated endovascularly (aneurysm coiled and aneurysm clipped) <24-h post-rupture and (2) external ventricular drainage placed <48-h post-rupture. Exclusion criteria were as follows: (1) CNS disease history, (2) CNS infection, and (3) systemic disease (diabetes mellitus, malignancy, and cirrhosis). On admission, clinical and hemorrhage severity were assessed by the Hunt and Hess (HH) grade, World Federation of Neurological Surgeons (WFNS) grade, and Fisher score (Dong et al., 2019). Patients received intravenous infusion of nimodipine for at least 7 days. Euvolemia was maintained, and hypotension was avoided with vasopressors (Dong et al., 2019). The end point was assessed at day 8. CSF samples were collected in sterile tubes or catheter and stored at −80°C. CSF samples of patients with normal pressure hydrocephalus (NPH) were as the experimental control because CSF is difficult to obtain from healthy individuals (Kwan et al., 2019).

### Rat SAH Model, Neurological Score, and CSF Collection

After approval by ethics committee of Shandong First Medical University, SAH models were produced in male Sprague–Dawley rats (12 weeks old, 320–350 g, Jinan Pengyue Laboratory Animal Center) by single blood injection model (Wu et al., 2017). Briefly, rats were deeply anesthetized (5% isoflurane) and then maintained (2% isoflurane) using a rodent ventilator (MatrixVMR). Non-heparinized autologous blood (0.3 ml) was injected into cisterna magna for 3 min using a 1-ml syringe with a 25-G needle under a stereotaxic apparatus. Sham-operated group underwent the same procedures except for injection of blood.

Three behavioral activity test of scoring system (Table 1) was performed at day 2 after SAH as previously described (Wu et al., 2017). Sequence of testing was randomized. Scoring was evaluated to record appetite, activity, and deficits blindly. About 50 μl of CSF was extracted from the cisterna magna (0.5 cm depth) with 1-ml syringe under a stereotaxic apparatus and then stored at −80°C (Zhang et al., 2020).

**Table 1:** Behavior scores.

**Category**	**Behavior**	**Score**
Appetite	Finished meal	0
	Left meal unfinished	1
	Scarcely ate	2
Activity	Active, walking, or standing	0
	Lying down, walk, and stand with some stimulations	1
	Almost always lying down	2
Deficits	No deficits	0
	Unstable walk	1
	Impossible to walk and stand	2

### Analysis of CSF HSP27 Concentration and Western Blot Analysis

The HSP27 of CSF was assayed using an enzyme linked immunosorbent assay (ELISA) Kit (ab108862, Abcam) according to the manufacturer's instruction and expressed in ng/ml. Western blot was conducted as previously described (Zhao et al., 2020). Briefly, total protein was extracted with a protein extraction kit (BC3710, Solarbio), which was supplemented with a protease inhibitor cocktail (P8340, Sigma), and analyzed by bicinchoninic acid (BCA) protein concentration kit (PC0020, Solarbio). About 20 μg of total protein was separated using sodium dodecyl sulfate (SDS) polyacrylamide gel electrophoresis and then electrotransferred onto nitrocellulose membrane. After blocking with 5% (w/v) non-fat milk, the membranes were incubated with anti-HSP27 (1:1,000, ab2790, Abcam), anti-active caspase-3 (1:1,000, ab2302, Abcam), anti-FLAG (1:1,000, F1804, Sigma), anti-phospho-MKK4 (Ser257/Thr261; 1:1,000, #9165, CST), anti-phospho-c-Jun N-terminal kinase (JNK) (Thr183/Tyr185; 1:1,000, #4688, CST), anti-phospho-c-Jun (Ser63; 1:1,000, #2361, CST), anti-MKK4 (1:1,000, #9152, CST), and β-actin (1:1,000, #4970, CST) at 4°C overnight and then incubated with anti-mouse immunoglobulin G-horseradish peroxidase (IgG-HRP; 1:3,000, #7076, CST) or anti-rabbit IgG-HRP (1:3,000, #7074, CST) linked antibody for 2 h at the room temperature. After washing with Tris-buffered saline with Tween 20 (TBST) buffer (T1081, Solarbio), the protein bands were visualized by using chemiluminescent substrate (#34577, Thermo Scientific) in a ChemiDoc^TM^ MP Imaging System (Bio-Rad) and quantified by Image J software.

### Adeno-Associated Virus, Peptides, and Intracerebroventricular Injection

The HSP27-overexpressing adeno-associated virus (AAV) and HSP27 shRNA (Table 2) were constructed by OBiO Technology (Shanghai, China). Full-length rat cDNA of HSP27 was cloned into the pAAV-CAG-3FLAG vector. AAV-HSP27-RNAi was constructed using the pAKD-CMV-EGFP-H1 vector, which contains a CMV promoter (driving EGFP) and a H1 promoter (driving shRNA expression). The sequence of HSP27 shRNA is 5′-GCTACATCTCTCGGTGCTTCA-3′ and 5′-GCCCAAAGCAGTCACACAATC-3′ as previously described (Stetler et al., 2008). AAV was produced by co-transfection (one AAV vector and two helper vectors) of HEK293T cells. At 72 h after transfection, cells were harvested and lysed using a freeze–thaw procedure. AAV of cell lysate was purified using a heparin-agarose column and concentrated using an ultrafiltration device. The virus titer was measured and shown in Table 2. The HSP27 peptides (Table 3) were synthesized by ChinaPeptides (Shanghai, China). For intracerebroventricular (i.c.v.) injection, rats received a single injection (0.8 mm posterior, 1.2 mm lateral, and 3.8 mm depth) of AAV (5 μl), TAT (0.3 mg, 10 μl), or TAT-HSP27_65−90_ (0.3 mg, 10 μl) with a 30-G needle of a 10-μl Hamilton syringe under a stereotaxic apparatus.

**Table 2:** Adeno-associated virus (AAV).

**Product**	**Serotype**	**Titer (pfu/ml)**	**Company**
pAAV-CAG-HSP27-3FLAG	AAV2/2	1.68 ×10^12^	OBiO technology
pAAV-CAG-3FLAG	AAV2/2	1.18 ×10^12^	
pAKD-CMV-EGFP-H1-shRNA-HSP27	AAV2/2	1.48 ×10^12^	
pAKD-CMV-EGFP-H1-shRNA-NC	AAV2/2	1.64 ×10^12^	

**Table 3:** HSP27 peptides.

**Product**	**Sequence**	**Purity**	**Company**
HSP27_1−30_	MTERRVPFSLLRSPSWEPFRDWYPAHSRLF	96.04%	China peptides
HSP27_31−60_	DQAFGVPRFPDEWSQWFSSAGWPGYVRPLP	96.28%	
HSP27_61−90_	AATAEGPAAVTLARPAFSRALNRQLSSGVS	97.41%	
HSP27_91−120_	EIRQTADRWRVSLDVNHFAPEELTVKTKEG	96.05%	
HSP27_65−90_	EGPAAVTLARPAFSRALNRQLSSGVS	95.80%	
TAT	YGRKKRRQRRR	96.75%	
TAT-HSP27_65−90_	YGRKKRRQRRREGPAAVTLARPAFSRALNRQLSSGVS	98.23%	

### Immunofluorescence and TUNEL Staining

Staining of slices was performed as previously described (Wang et al., 2019). Briefly, rats were deeply anesthetized and perfused transcardially with ice-cold 4% paraformaldehyde. Rat's brain was post-fixed with 4% paraformaldehyde for 12 h, dehydrated with 30% sucrose/phosphate-buffered saline (PBS) at 4°C for 3 days, and then cut into 10-μm thickness coronal slices (−2.5 to −5 mm from bregma) using a Leica cryostat microtome. Coronal slices were permeabilized with 0.5% Triton X-100 and blocked with 5% goat serum, incubated with anti-NeuN rabbit antibody (1:200, #12943, CST), anti-NeuN mouse antibody (1:200, #94403, CST), anti-HSP27 (1:200, ab2790, Abcam), anti-Iba-1 (1:200, #19741, Wako) primary antibody at 4°C overnight and then incubated with the Anti-Mouse IgG-TRITC (1:100, T5393, Sigma) or Anti-Rabbit IgG-FITC (1:100, F9887, Sigma) at room temperature for 2 h. Terminal deoxynucleotidyl transferase-mediated dUTP nick end labeling (TUNEL) staining was assayed using *in situ* Cell Death Detection Kit with Fluorescein (11684795910, Roche) according to the manufacturer's instruction. Images were captured using a fluorescence microscope (BX51, Olympus) and analyzed with Image J software.

### Hemolysate Treatment and TUNEL Assay on Primary Cortical Neurons

Primary cortical neurons were obtained from E18 rat embryos as previously described (Zhang et al., 2018). Briefly, the dissociated neurons (10^6^ cells) were plated in 60 mm dish with neurobasal medium [2% B27 (Gibco), 1% L-glutamine (Gibco), 0.3% D-glucose (Sigma), and 1% fetal bovine serum (Gibco)] and fed with fresh medium every 3 days. Hemolysate-induced cortical neuron death model was produced as described previously (Li et al., 2017). Briefly, hemolysate was prepared from mouse arterial whole blood by freezing and stored at −80°C. In order to induce neuronal death, hemolysate in medium (1:50) was used to stimulate for 24 h. Neurons were treated with hemolysate in medium (1:50) or plus indicated HSP27 peptides (0.03 mg/ml, Table 3) for 24 h. Cell viability was measured with Cell Counting Kit-8 (CCK-8, Dojindo) according to the manufacturer's instruction. Cell apoptosis was assayed with TUNEL bright-red apoptosis kit (A113, Vazyme) according to the manufacturer's instruction. Images of cultured neurons were captured under a phase contrast microscope (phase contrast, ×200). Pictures of TUNEL staining were captured under a fluorescence microscope (red, ×200). In addition, the total protein of neurons was extracted and then analyzed by Western blot.

### Statistical Analysis

GraphPad Prism 6 was used to perform statistical analyses. All results were expressed as means ± standard deviation (SD). Data of figure were analyzed using one-way analysis of variance (ANOVA) followed by the Bonferroni's multiple comparisons test and the two-tailed *t*-test. *p* < 0.05 considered statistically significant.

## Results

### HSP27 Level of CSF in Patients With SAH

**Figure 1:**
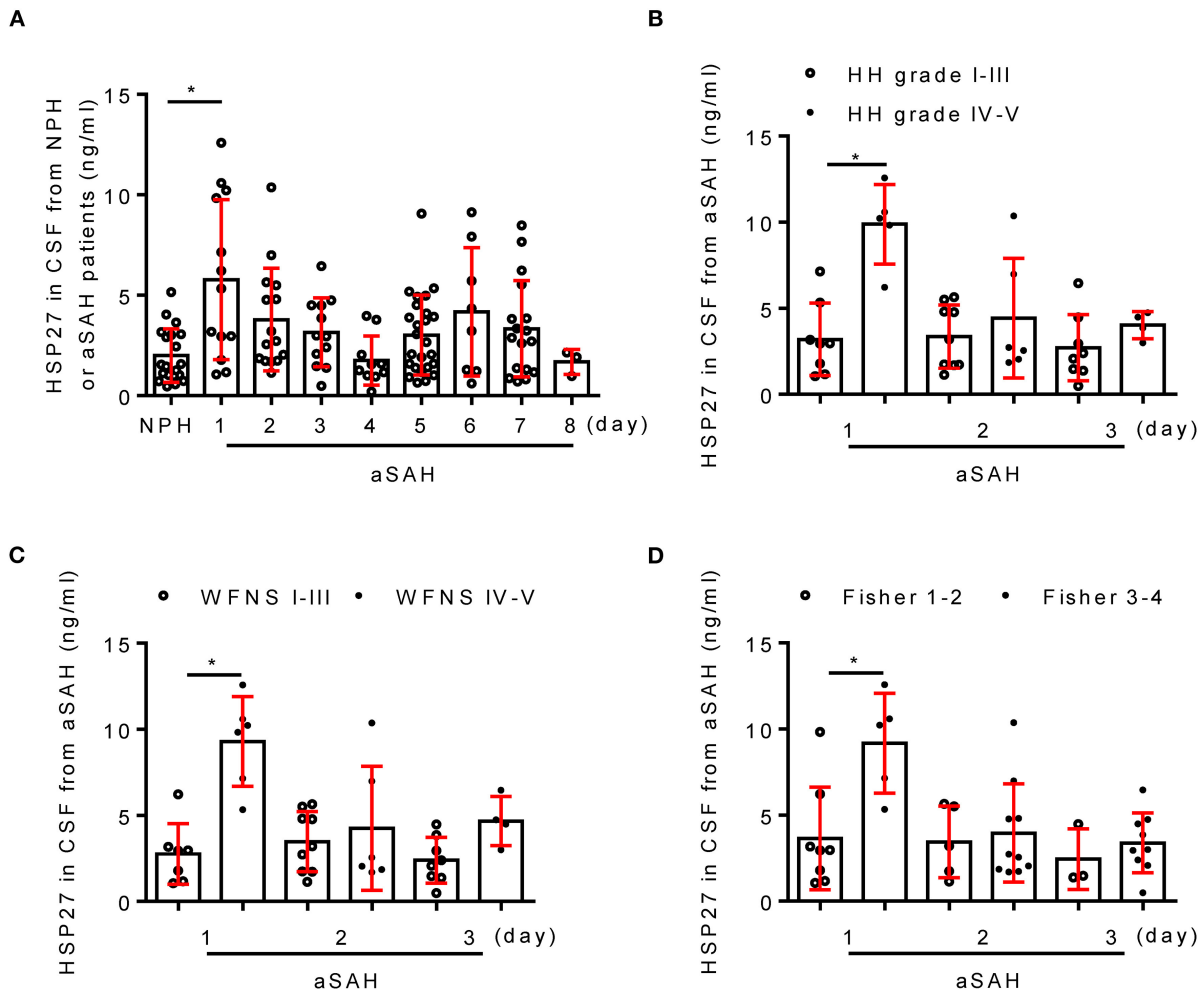
Change of CSF HSP27 level in patients with aSAH. **(A)** Concentration of CSF HSP27 of patients with NPH and aSAH is measured at indicated time with HSP27 ELISA Kit. The histogram shows CSF HSP27 level of patients with aSAH with HH grades I–III and IV–V **(B)**, WFNS grades I–III and IV–V **(C)**, Fisher score 1–2 and 3–4 **(D)**. The values are expressed in ng/ml. Data are mean ± SD, *p < 0.05, ANOVA with Bonferroni's multiple comparisons test.

Table 4 shows the clinical characteristics of patients with aSAH and NPH. There was no significant difference in age and gender between two groups (*p* > 0.05). In patients with aSAH, HH grade, WFNS grade, and modified Fisher score (on admission) were 2.6 ± 1.0, 2.4 ± 1.5, and 2.9 ± 0.7, respectively. Next, the temporal course of HSP27 level (day 1 to 8) following aSAH is depicted in Figure 1. HSP27 level of CSF on day 1 was significantly increased in comparison to that in patients with NPH. HSP27 level of day 2 to 4 was gradually decreased in comparison to day 1 (Figure 1A). Furthermore, there was a significant difference in HSP27 level (day 1) between patients with aSAH with HH grade of I–III vs. IV–V (Figure 1B), WFNS grades of I–III vs. IV–V (Figure 1C), and Fisher scores of 1–2 vs. 3–4 (Figure 1D).

**Table 4:** Characteristics for patients.

	**aSAH**	***NPH***
	**(*n* = 73)**	**(*n* = 20)**
Demographics		
Age, years	56.5 ± 8.0	51.8 ± 9.0
Gender, female	47 (64.3)	12 (60)
Clinical status on admission		
Hunt and Hess grade	2.6 ± 1.0	—
WFNS grade	2.4 ± 1.5	—
Fisher score	2.9 ± 0.7	—
Aneurysm location		
Internal carotid artery	18 (24.6)	—
Middle cerebral artery	16 (21.9)	—
Anterior communicating artery	30 (41.1)	—
Others	9 (12.3)	—
Aneurysm treatment		
Coiling	61 (83.5)	—
Clipping	12 (16.5)	—

*Values were expressed as mean ± SD or numbers (% of total)*.

### HSP27 Expression Is First Increased and Then Declined After Rat SAH

**Figure 2:**
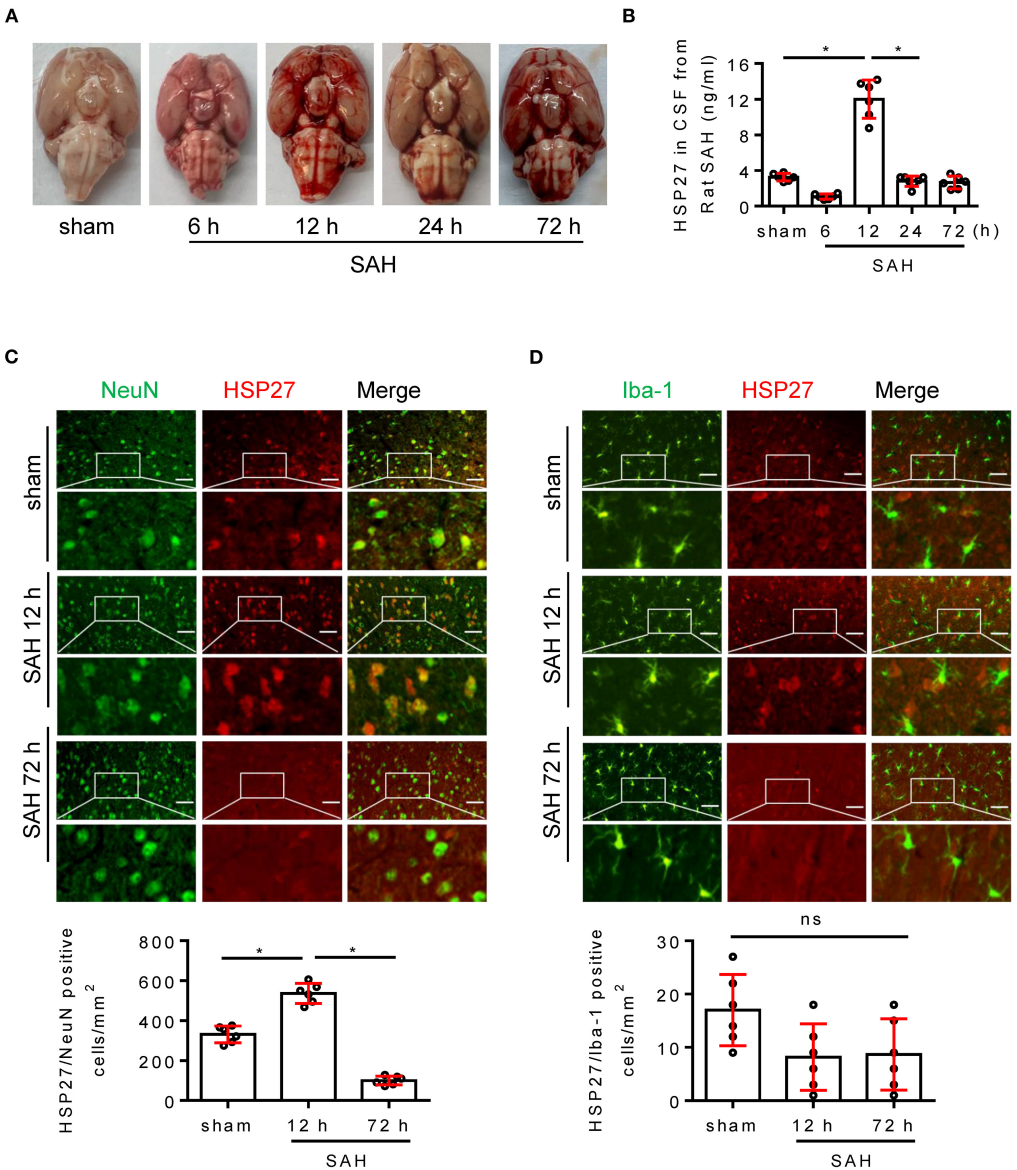
Change of HSP27 level in rat SAH model. **(A)** Representative images of rat brain at indicated time from the sham and SAH groups. **(B)** Samples of CSF was collected (6, 12, 72 h, *n* = 6; 24 h, *n* = 7), HSP27 concentration was measured with HSP27 ELISA Kit and expressed in ng/ml. **(C,D)** Coronal sections from the sham and SAH group reperfusion (12 and 72 h) subjected to immunostaining for the neuronal marker NeuN (green) or macrophages/microglia marker Iba-1 (green) and HSP27 (red) in the basal cortex. Quantification was performed by counting the HSP27/NeuN or HSP27/Iba-1 positive cells per mm^2^ region in the basal cortex, *n* = 6, scale bar = 50 μm. Data are mean ± SD, *p < 0.05, ANOVA with Bonferroni's multiple comparisons test.

Next, we evaluated the expression of HSP27 in brain using the rat SAH model. The calculated mortality rate at 72 h is given in Table 5. Blood clots were observed on the circle of Willis in SAH groups (Figure 2A). ELISA showed that HSP27 level of CSF was obviously increased at 12 h in comparison to that in sham group and then declined significantly (Figure 2B). In immunofluorescence staining, HSP27 can be co-located with NeuN (a marker for neuron) (Figure 2C), whereas rarely co-located with Iba-1 (a marker for macrophages/microglia) (Figure 2D). Moreover, HSP27 staining was significantly increased at 12 h compared with that of the sham group, whereas obviously declined at 72 h (Figures 2C,D). These results indicate that expression of HSP27 is first increased and then declined in rat SAH.

**Table 5:** Mortality rate.

**Groups**	**Endpoint**	**Mortality rate**	**Included (*n*)**
**Experiment 1 (ELISA, IF)**	72 h		
Sham		0.0% (0/6)	6
6 h		0.0% (0/8)	6
12 h		0.0% (0/8)	6
24 h		12.5% (1/8)	7
72 h		25.0% (2/8)	6
**Experiment 2 (Behavior, IF, WB, TUNEL)**	48 h		
Sham		0.0% (0/12)	12
SAH + vehicle		25.0% (4/16)	12
SAH + NC		18.7% (3/16)	13
SAH + shRNA		31.3% (5/16)	11
**Experiment 3 (Behavior, IF, WB, TUNEL)**	48 h		
Sham		0.0% (0/12)	12
SAH + vehicle		25.0% (4/16)	12
SAH + Con		25.0% (4/16)	12
SAH + HSP27		12.5% (2/16)	14
**Experiment 4 (Behavior, IF, WB, TUNEL)**	48 h		
Sham		0.0% (0/12)	12
SAH + vehicle		31.25% (5/16)	11
SAH + TAT		18.7% (3/16)	13
SAH + TAT-HSP27		12.5% (2/16)	14

### Knockdown of HSP27 Deteriorates Neurological Deficit After Rat SAH

**Figure 3:**
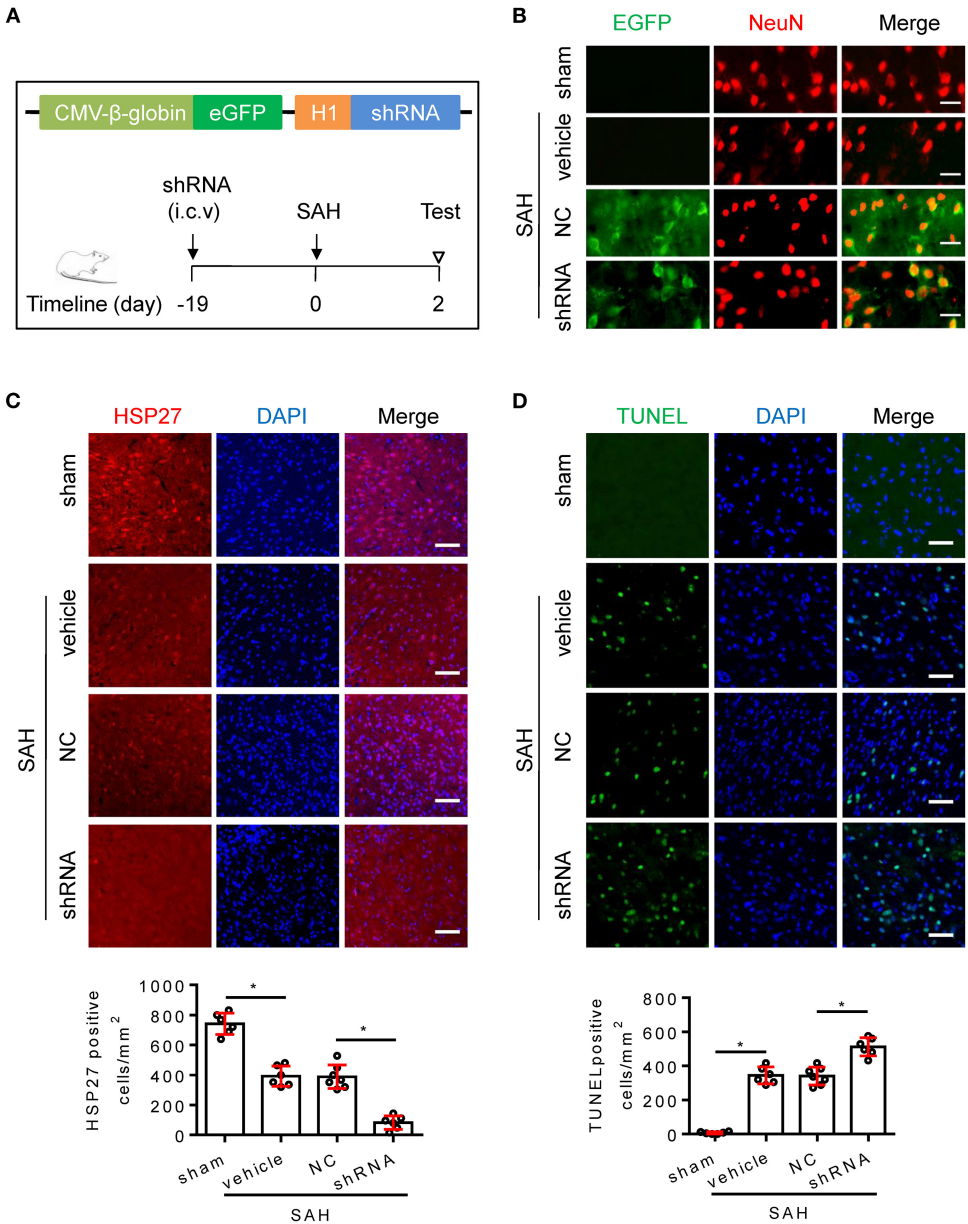
AAV-shRNA targeting HSP27 increased cell apoptosis after SAH in rats. **(A)** Schematic of AAV vector encoding shRNA and experimental design. **(B–D)** Coronal sections from the sham (*n* = 6), SAH + vehicle (*n* = 6), SAH + negative control of shRNA (SAH + NC, *n* = 7), and SAH + HSP27 shRNA (SAH + shRNA, *n* = 6) group reperfusion on day 2 after SAH, subjected to immunostaining for the neuronal marker NeuN (red) or HSP27 (red) or TUNEL (green) in the basal cortex. **(B)** Representative images showing AAV-eGFP-shRNA expression (indicated by EGFP, green) in the neurons (NeuN staining, red) in the basal cortex, scale bar = 50 μm. **(C,D)** Quantification was performed by counting the HSP27 or TUNEL positive cells per mm^2^ region in the basal cortex, scale bar = 50 μm. Data are mean ± SD, *p < 0.05, ANOVA with Bonferroni's multiple comparisons test.

**Figure 4:**
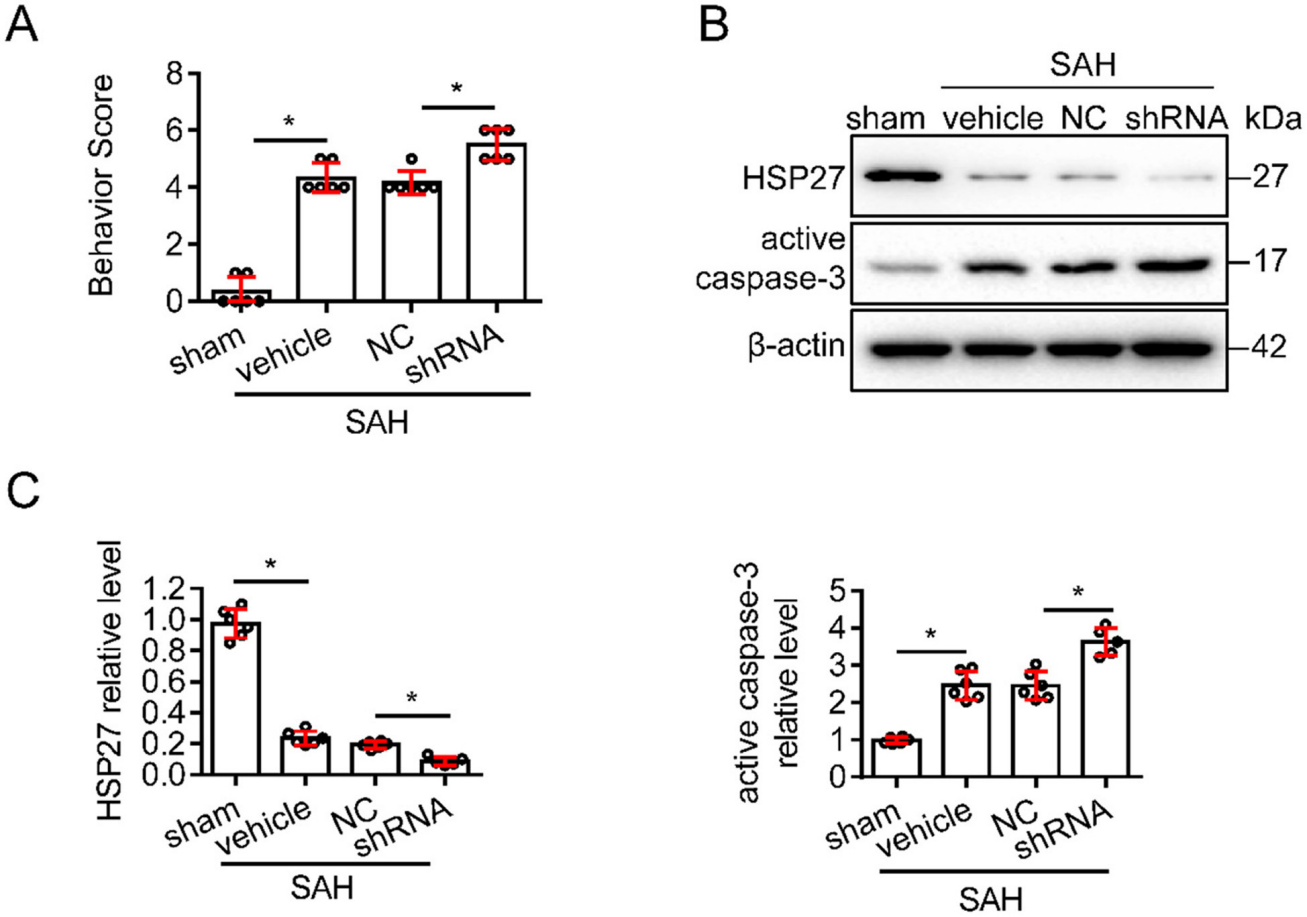
AAV-shRNA targeting HSP27 deteriorated neurological deficit after SAH in rats. **(A)** Representative images of rat brain on day 2 after surgery for the sham, SAH + vehicle, SAH + NC, and SAH + shRNA groups. **(B)** Behavior scores of each group were assessed at 48 h, *n* = 6. **(C)** The basal cortex was collected on day 2 following SAH from the sham (*n* = 6), SAH + vehicle (*n* = 6), SAH + NC (*n* = 6), and SAH + shRNA (*n* = 5) groups; homogenates were blotted with anti-HSP27, anti-active caspase-3, and anti-β-actin, quantification of optical density was normalized to sham group. Data are mean ± SD, *p < 0.05, ANOVA with Bonferroni's multiple comparisons test.

To confirm whether endogenous HSP27 has effect on neurological function, rat SAH was subjected to HSP27 knockdown using AAV-eGFP-shRNA (Figure 3A). The calculated mortality rate at 48 h is given in Table 5. After microinjection into the lateral ventricle of rat (Figure 3A), AAV-eGFP-shRNA effectively infected the basal cortex, produced considerable expression of eGFP (Figure 3B), and significantly decreased expression of HSP27 (Figures 3C, 4C), suggesting an effective knockdown effect. Statistical results of TUNEL staining showed that numerous TUNEL positive cortical cells significantly increased in SAH + HSP27 shRNA group as compared with that of SAH + negative control of shRNA group on day 2 after SAH (Figure 3D). Moreover, the behavior scores in SAH + HSP27 shRNA group were significantly increased when compared to SAH + negative control of shRNA group (Figures 4A,B), suggesting that HSP27 shRNA worsens neurological function after SAH. Western blot analysis showed that the activation of caspase-3 was significantly increased in SAH + HSP27 shRNA group as compared to SAH + negative control of shRNA group (Figure 4C). These results indicate that knockdown of endogenous HSP27 increases cell apoptosis and deteriorates neurological deficit in rat SAH.

### HSP27 Overexpression Attenuates Neurological Deficit and Cortical Apoptosis After Rat SAH

**Figure 5:**
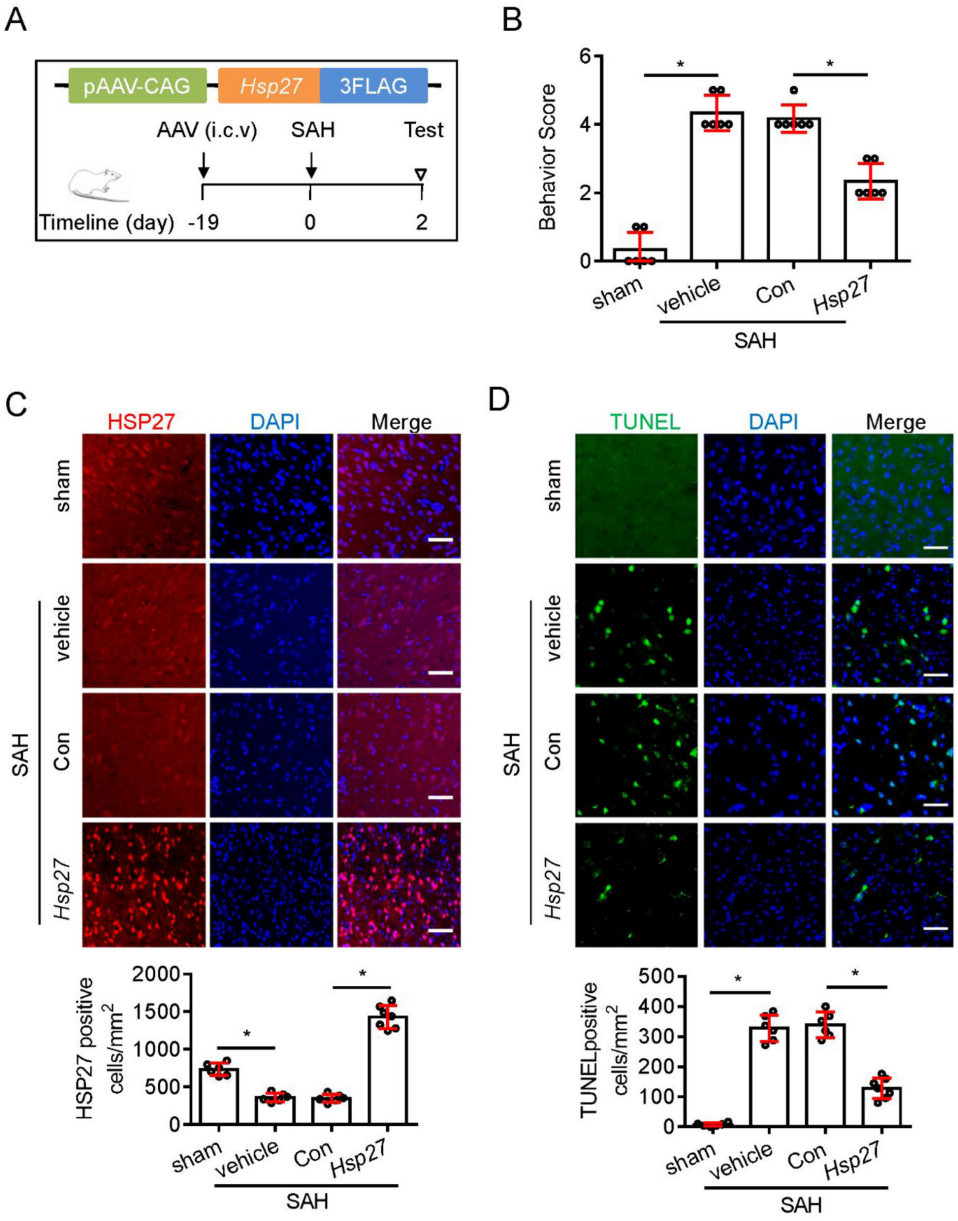
HSP27 overexpression attenuated neurological deficit and cell apoptosis after SAH in rats. **(A)** Schematic of AAV vector encoding HSP27 and experimental design. Representative images of rat brain on day 2 after surgery for the sham, SAH + vehicle, SAH + AAV vector (SAH + Con), and SAH + AAV vector encoding HSP27 (SAH + HSP27) group. **(B)** Behavior scores of each group were assessed at 48 h, *n* = 6. **(C,D)** Coronal sections from the sham (*n* = 6), SAH + vehicle (*n* = 6), SAH + Con (*n* = 6), and SAH + HSP27 (*n* = 7) group reperfusion on day 2 after SAH, subjected to immunostaining for the HSP27 (red) or TUNEL (green) in the basal cortex. Quantification was performed by counting the HSP27 or TUNEL positive cells per mm^2^ region in the basal cortex, scale bar = 50 μm. Data are mean ± SD, *p < 0.05, ANOVA with Bonferroni's multiple comparisons test.

**Figure 6:**
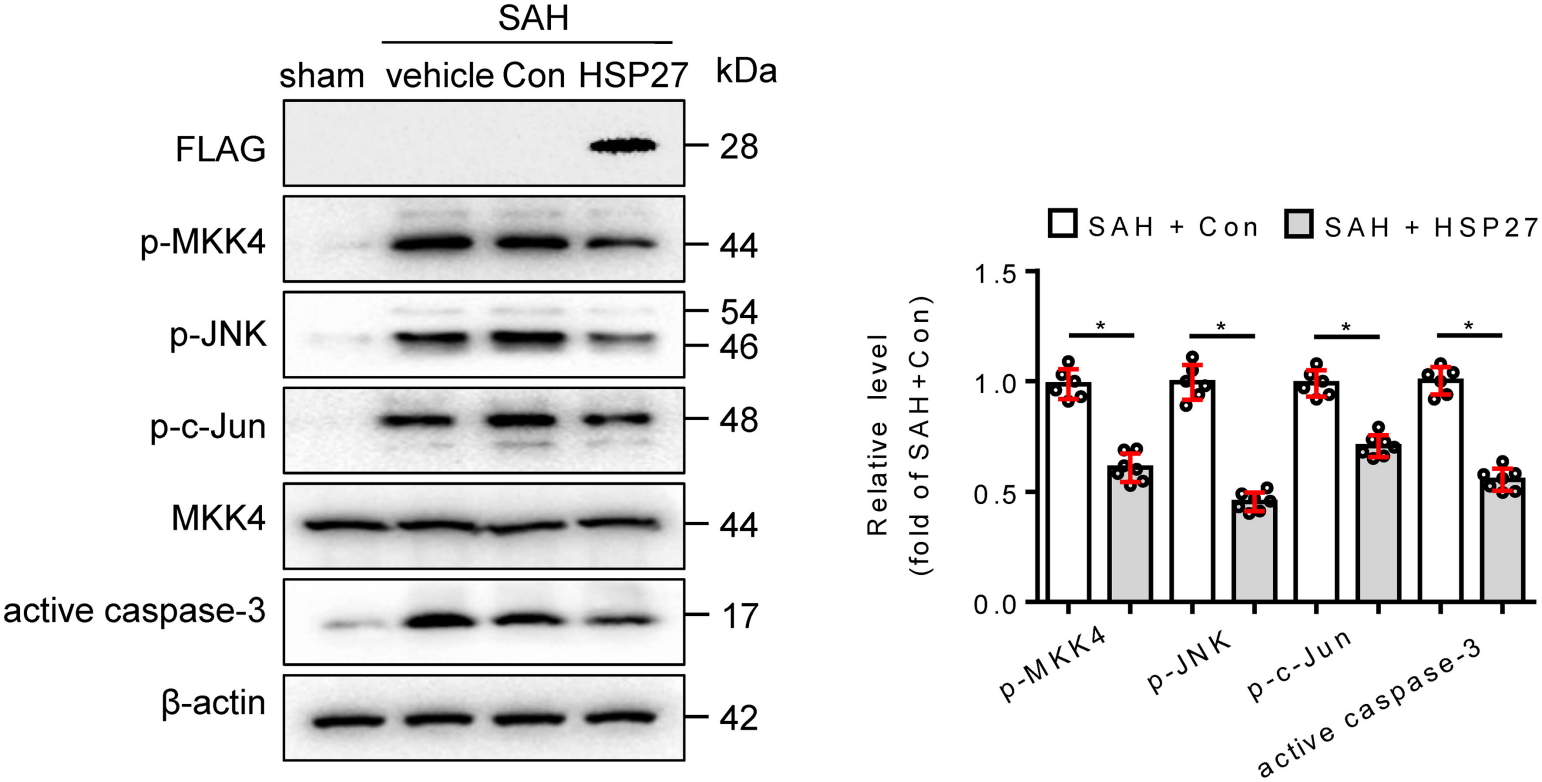
Effect of HSP27 overexpression on the activation of MKK4, JNK, c-Jun, and caspase-3 on day 2 after SAH. The basal cortex was collected on day 2 following SAH from the sham (*n* = 6), SAH + vehicle (*n* = 6), SAH + Con (*n* = 6), and SAH + HSP27 (*n* = 7) groups; homogenates were blotted with anti-FLAG, anti-phospho-MKK4, anti-phospho-JNK, anti-phospho-c-Jun, anti-MKK4, anti-active caspase-3, and anti-β-actin; and quantification of optical density was normalized to sham group. Data are mean ± SD, *p < 0.05, the two-tailed *t*-test.

To test whether HSP27 overexpression has effect on neurological function, rat SAH was infected with AAV-HSP27-3FLAG (Figure 5A), which expresses full-length HSP27. The calculated mortality rate at 48 h is given in Table 5. The behavior score analysis showed that behavior scores in SAH + AAV vector encoding HSP27 group were significantly decreased when compared to SAH + AAV vector group (Figure 5B), suggesting an improvement effect. After microinjection into the lateral ventricle of rat, immunofluorescence staining showed that AAV-HSP27-3FLAG obviously increased expression of HSP27 in the basal cortex (Figure 5C). Western blot confirmed the increased HSP27 protein expression by detecting FLAG expression (Figure 6). Moreover, statistical results of TUNEL staining showed that numerous TUNEL positive cortical cells significantly decreased in SAH + AAV vector encoding HSP27 group as compared with that of SAH + AAV vector group on day 2 after SAH (Figure 5D). Previous review showed that HSP27 exerts an anti-apoptosis role by inhibiting MKK/JNK cell death signal, and or mitochondria-related pro-apoptotic factors (Shan et al., 2021). Thus, we further examined the activity of MKK/JNK and caspase-3 by Western blot. Data showed that the phosphorylation of MKK4, JNK, and c-Jun and the activation of caspase-3 were obviously increased in SAH + vehicle and SAH + AAV vector group, whereas significantly decreased in SAH + AAV vector encoding HSP27 group (Figure 6). These results indicate that HSP27 overexpression attenuated cortical cell apoptosis and neurological deficit in rat SAH.

### Effect of HSP27 Peptides on Hemolysate-Induced Cell Apoptosis in Cultured Cortical Neurons

**Figure 7:**
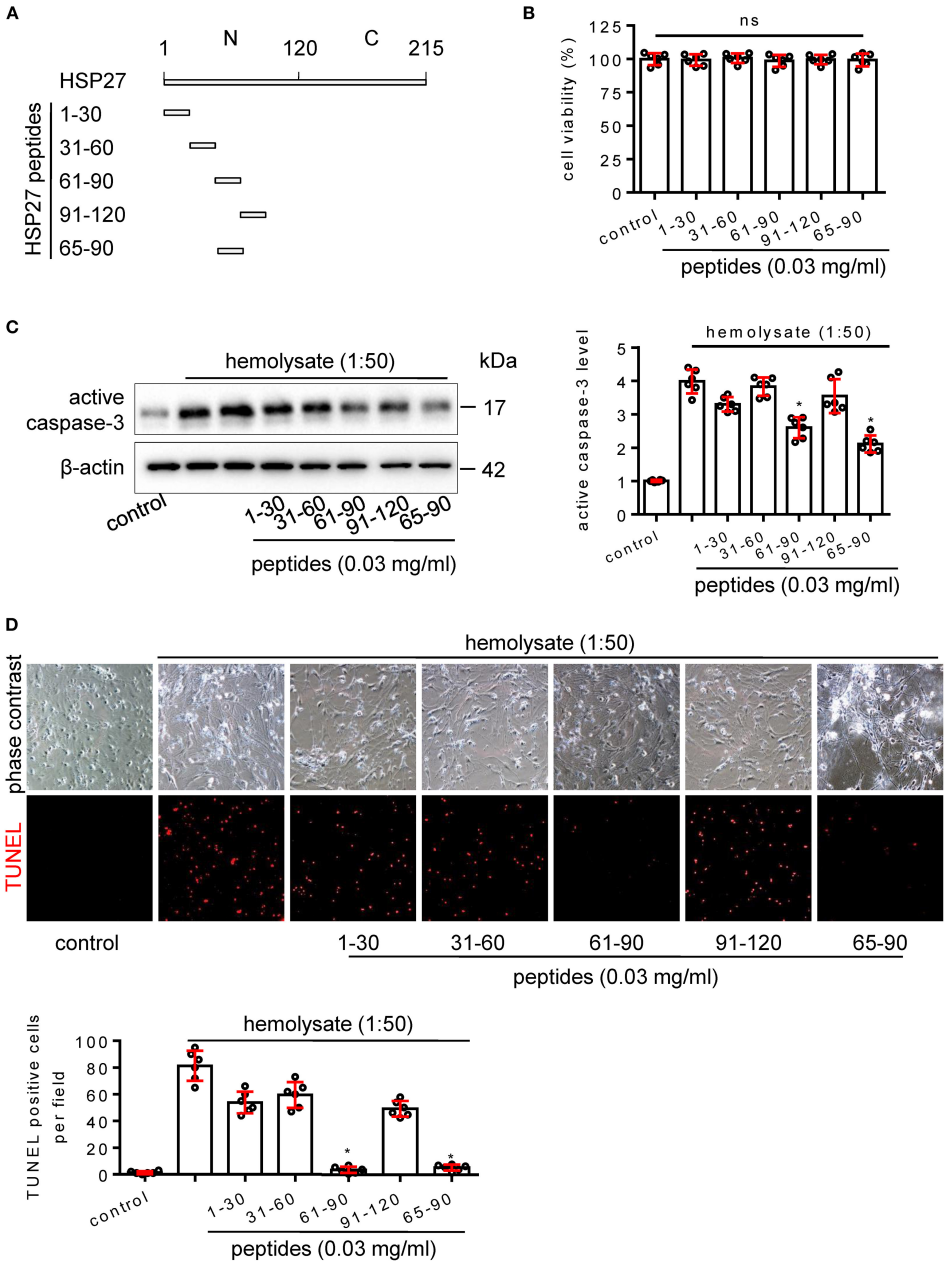
Effect of HSP27 peptides on hemolysate-induced cell apoptosis in primary cortical neurons. **(A)** Schematic representation of various HSP27 peptides. **(B)** Hsp27 peptides have no effect on cell viability in primary cortical neurons; Cortical neurons were treated with indicated HSP27 peptides (0.03 mg/ml) in medium (1:50) for 24 h. Cell viability was measured with Cell Counting Kit-8 (CCK-8) and normalized to control. **(C, D)** Cortical neurons were treated with hemolysate in medium (1:50) or plus indicated HSP27 peptides (0.03 mg/ml) for 24 h. **(C)** Active caspase-3 levels in each group were detected by Western blot, β-actin serves as a control, and quantification of optical density was normalized to control. **(D)** Representative images of cortical neurons (phase contrast, ×200) and TUNEL staining (red, ×200), and quantification of TUNEL-positive cells from each group was performed. Data are mean ± SD, *n* = 6, *p < 0.05 vs. hemolysate treatment, ANOVA with Bonferroni's multiple comparisons test.

N-terminal region of HSP27 is composed of 1–120 amino acids and proved to be a necessary domain for neuroprotection (Stetler et al., 2008). So, we designed and synthesized peptides from the N-terminal 1–120 amino acids of HSP27 (Figure 7A), which had no effect on cell activity in cultured cortical neuron (Figure 7B), aiming to find the key peptide that affects cell apoptosis. Using a hemolysate-induced cortical neuron death model, Western blot and TUNEL staining showed that HSP27_61−90_ and HSP27_65−90_ peptides, but not HSP27_1−30_, HSP27_31−60_, and HSP27_91−120_ peptides, can effectively inhibit hemolysate-induced the increased the activation of caspase-3 (Figure 7C) and reduce hemolysate-elevated the number of TUNEL positive cells (Figure 7D). These results suggest that the N-terminal 65–90 amino acids of HSP27 are the key area for affecting cell apoptosis.

### TAT-HSP27_65–90_ Peptide Attenuates Neurological Deficit and Cortical Apoptosis After Rat SAH

**Figure 8:**
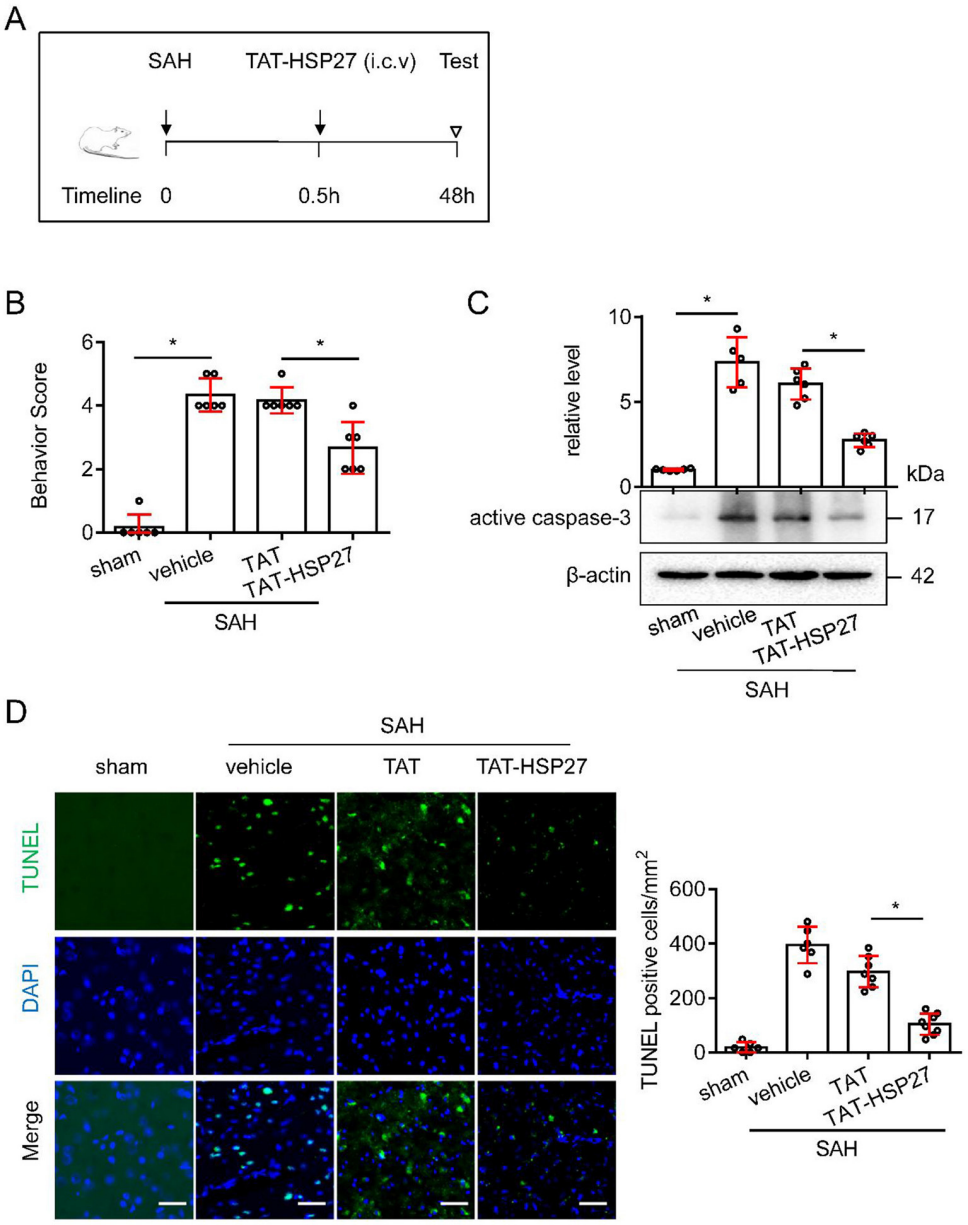
TAT- HSP27_65−90_ peptide attenuated neurological deficits and cell apoptosis after SAH in rats. **(A)** Experimental design; representative images of rat brain on day 2 after surgery for the sham, SAH + vehicle, SAH + TAT peptide (SAH + TAT), and SAH + TAT-HSP27_65−90_ peptide (SAH + TAT-HSP27) groups. **(B)** Behavior scores of each group were assessed at 48 h, *n* = 6. **(C)** The basal cortex was collected on day 2 following SAH from the sham (*n* = 6), SAH + vehicle (*n* = 5), SAH + TAT (*n* = 6), and SAH + TAT-HSP27 (*n* = 6) groups; homogenates were blotted with anti-active caspase-3 and anti-β-actin, and quantification of optical density was normalized to sham group. **(D)** Coronal sections from the sham (*n* = 6), SAH + vehicle (*n* = 6), SAH + TAT (*n* = 7), and SAH + TAT-HSP27 (*n* = 8) group reperfusion on day 2 after SAH, subjected to immunostaining for the TUNEL (green) in the basal cortex. Quantification was performed by counting the TUNEL positive cells per mm^2^ region in the basal cortex, scale bar = 50 μm. Data are mean ± SD, *p < 0.05 vs. hemolysate treatment, ANOVA with Bonferroni's multiple comparisons test.

Furthermore, we investigated whether TAT-HSP27_65−90_ peptide could improve neurological deficit in rat SAH. The calculated mortality rate at 48 h is given in Table 5. As expected, after microinjection into the lateral ventricle of rat SAH (Figure 8A), TAT-HSP27_65−90_ significantly decreased the behavior score as compared with the TAT-treated SAH group (Figure 8B), suggesting that TAT-HSP27_65−90_ treatment attenuates SAH-induced neurological deficits. In addition, Western blot analysis suggested that the vehicle or TAT-treated SAH group showed upregulation of active caspase-3, which was attenuated by TAT-HSP27_65−90_ treatment (Figure 8C). Furthermore, TUNEL staining suggested that TUNEL-positive cells of the basal cortex were significantly decreased at 48 h in the TAT-HSP27_65−90_-treated SAH group when compared to the TAT-treated SAH group (Figure 8D), indicating TAT-HSP27_65−90_ reduced cortical cell apoptosis after SAH. There are two commonly used SAH models: blood injection model and endovascular puncture model (Kooijman et al., 2014). Later, we explored the effect of TAT-HSP27_65−90_ on neurological deficit and cell apoptosis in the endovascular puncture rat SAH model. There was no statistical difference in SAH grading score among the vehicle, TAT, or TAT-HSP27_65−90_-treated SAH group (Supplementary Figure S1A). TAT-HSP27_65−90_ treatment significantly increased the average modified Garcia score as compared with the TAT-treated SAH group (Supplementary Figure S1B), suggesting that TAT-HSP27_65−90_ treatment improved neurological deficits after SAH. Moreover, NeuN staining suggested that NeuN-positive neurons of the hippocampal CA1 region were significantly increased at 24 h in the TAT-HSP27_65−90_-treated SAH group in comparison with that of the TAT-treated SAH group (Supplementary Figure S1C). TUNEL staining suggested that TUNEL-positive cells of the basal cortex were significantly decreased at 24 h in the TAT-HSP27_65−90_-treated SAH group when compared to the TAT-treated SAH group (Supplementary Figure S1C), indicating TAT-HSP27_65−90_ reduced cortical cell death after SAH in the endovascular puncture model.

## Discussion

The main findings of this study are as follows: change of HSP27 level in CSF from patients with aSAH; expression of HSP27 is first increased and then declined after SAH in rats; and knockdown of HSP27 deteriorated neurological deficit, whereas overexpression of HSP27 confers neuroprotection after SAH in rats. TAT-HSP27_65−90_ peptide effectively reduces hemolysate-induced cell apoptosis on cultured cortical neurons and attenuates neurological deficit after SAH in rats.

Since intracerebral microdialysis allows *in vivo* sampling of interstitial fluid, it is used to continuously monitor the neurochemical metabolism of the damaged brain after aSAH, but this method is limited to the injured tissue around the probe (Unterberg et al., 2001; Helbok et al., 2015). Physiologically, CSF is secreted in the choroid plexus and recirculates through the cerebral ventricles and subarachnoid space, interchanging with interstitial fluid, which is thought to play a role in clearance of solutes and metabolic waste products from the brain (Iliff et al., 2013; Xie et al., 2013). Compared to intracerebral microdialysis, CSF analysis may reflect a more general picture of brain injury. CSF analysis basing on lumbar puncture is cornerstone for aSAH diagnosis, which means that changes of proteins can be detected quickly and conveniently to clarify the correlation between these changes and SAH pathology (Wasik et al., 2017; Papa et al., 2018; Kwan et al., 2019). To our knowledge, this study is the first to report the change of CSF HSP27 in patients with aSAH as compared with patients with NPH, showing a trend of increasing first and then decreasing in the early days. Our findings show that CSF HSP27 is obviously increased on day 1, and related to the grades of HH, WFNS, and Fisher score, which means that early higher level of HSP27 is related to clinical and hematological severity. CSF HSP27 is increased second in about 6 days, whether it is related to the occurrence of complications after SAH needs further study. Using the rat SAH model, we observed that CSF HSP27 decreased at 6 h, increased significantly at 12 h, and then significantly decreased at 24 and 72 h. The change trend of CSF HSP27 in EBI is similar to that of patients with aSAH in the early days. There have been two previous studies relating to HSP27 expression in SAH. The first study involved a rat SAH model where HSP27 expression was observed in the cerebral arteries at 48 h post-ictus, the overall expression of HSP27 almost unchanged, whereas the phosphorylated isoforms of HSP27 increased (Macomson et al., 2002). In the second study, the protein level of HSP27 was markedly decreased at 0.5 h and significantly elevated in brain stem after rat SAH (Satoh et al., 2003). Interestingly, our immunofluorescence results showed that the change of HSP27 expression mainly occurred in neurons rather than in macrophages/microglia after SAH. Based on the results of animal experiments, the change trend of HSP27 in CSF and the basal cortex is similar. We speculate that the change trend of CSF HSP27 may reflect the change of HSP27 in human brain tissue after aSAH.

Although the change of HSP27 may be an endogenous response and a possible protective mechanism against brain injury following SAH, this has not been directly tested with HSP27 knockdown or overexpression. Our data showed that worsening neurological deficit was observed in rat SAH after knockdown of endogenous HSP27 using the previously reported shRNA. However, AAV-mediated overexpression of HSP27 attenuated SAH-induced neurological deficits and cell apoptosis in the basal cortex of rats. It has been shown that overexpression of HSP27 provides neuroprotection in cerebral ischemia mice model, which indicated the mechanism involving the inhibition of mitochondrial cell death signaling (Stetler et al., 2008, 2012; Leak et al., 2013; Shi et al., 2017). We observed that the knockdown of endogenous HSP27 increased activation of pro-apoptotic molecular caspase-3, whereas virus-mediated overexpression of HSP27 inhibits activation of caspase-3 after SAH in rats. Indeed, HSP27 is reported to inhibit cell apoptosis by reducing cytochrome c release from mitochondria (Garrido et al., 1999) and consequently downregulating cleaved caspase-3 (Garrido et al., 1999; Bruey et al., 2000). Moreover, HSP27 can directly bind to the prodomain of caspase-3 and attenuates its proteolytic activation (Pandey et al., 2000; Voss et al., 2007). Our data also showed that overexpression of HSP27 attenuates SAH-elevated kinase phosphorylation of MKK4, JNK, and c-Jun in rats. Some reports have found that HSP27 inhibits cell apoptosis by hindering MKK/JNK cell death signal pathway induced by oxidative stress or ischemia (Stetler et al., 2008, 2012). Because oxidative stress and delayed cerebral ischemia are the key factors of cell apoptosis after SAH (Sabri et al., 2013; Yang et al., 2017), we speculate that overexpression of HSP27 maybe reduce cell apoptosis through inhibiting caspase activity and phosphorylation of MKK4 and JNK after SAH in rats. In non-phosphorylated form, HSP27 is an ATP-independent molecular chaperone and exists as the high molecular weight oligomeric; upon different stimuli, HSP27 is phosphorylated at Ser15, Ser78, and Ser82 by several protein kinases, and phosphorylation of HSP27 changes its conformation, which shifts from the large oligomers to dimers and monomers (Stetler et al., 2009; Sharp et al., 2013). Whether HSP27 phosphorylation occurs and subsequently mediates JNK phosphorylation in EBI after SAH needs to be verified in the following studies.

In addition to the overexpression of HSP27 that provides neuroprotection, the synthetic mimic peptide where transduced PEP-1-HSP27 peptide, a fusion peptide consisting of the PEP-1 peptide and human HSP27, can protect against ischemic injury in a gerbil animal model (An et al., 2008); intravenous injection of TAT-HSP27 peptide, a fusion peptide consisting of TAT and human HSP27 ameliorated ischemia/reperfusion-induced neurological deficits in mice (Shi et al., 2017). The structure of HSP27 includes the N-terminal domain containing three serine phosphorylation sites that regulates the reconfiguration of oligomeric function and structure, and the C-terminal domain containing β-pleated sheets that functions protein–protein interactions (Stetler et al., 2009; Sharp et al., 2013). The N-terminal region of HSP27 proved to be a necessary domain for neuroprotection *in vitro* ischemia (Stetler et al., 2008). So, we synthesized peptides from the N-terminal region of HSP27 and found that the HSP27_65−90_ peptide reduces cortical neuronal death in an *in vitro* hemolysate-damaged cortical neuron model (Li et al., 2017), which contains multiple components and mimics the pathophysiological scenario of SAH observed *in vivo* (Zhou et al., 2007). Generally, it is believed that SAH causes neuronal death in the basal cortex exposed to bloody CSF (Park et al., 2004). TAT protein transduction domain can facilitate the delivery of proteins or peptides without cell type specificity, its fusion peptide can be delivered into the brain parenchyma after systemic injection (Cao et al., 2002; Wang et al., 2019). Similar to overexpression of HSP27, we observed that i.c.v. injection of TAT-HSP27_65−90_ peptide provides neuroprotection, suppresses mitochondrial cell apoptosis signaling of active caspase-3, and attenuates cellular apoptosis in the basal cortex after SAH in rats. There are several limitations in this study. First, we utilized young male rats to make the SAH model, whereas aSAH mostly occurs in middle-aged and elderly women (Duan et al., 2018). Second, we studied the overexpression or knockdown of HSP27 on neurological deficit in blood injection SAH model, which is fairly reproducible mild SAH model developed by injecting a fixed amount of blood into the subarachnoid space (Prunell et al., 2003).

## Conclusion

In conclusion, the present study shows that early CSF level of HSP27 is related to clinical and hematological severity in patients with aSAH. Expression of HSP27 is first increased and then declined; overexpression of HSP27, but not knockdown of HSP27, confers neuroprotection after SAH in rats. TAT-HSP27_65−90_ peptide can effectively inhibit neuronal death in an *in vitro* hemolysate-damaged cortical neuron model, and thus attenuated neurological deficit after SAH in rats.

## Data Availability Statement

The original contributions presented in the study are included in the article/Supplementary Material, further inquiries can be directed to the corresponding author/s.

## Ethics Statement

The studies involving human participants were reviewed and approved by Ethical Committee of Shandong Provincial Hospital. The patients/participants provided their written informed consent to participate in this study. The animal study was reviewed and approved by Ethics Committee of Shandong First Medical University.

## Author Contributions

J-XH, B-lS, and Z-yZ designed experiments, analyzed the results, and wrote the manuscript. X-yZ, J-yS, W-qW, S-xL, H-xL, M-fY, and HY performed experiments. All authors read and approved the final manuscript.

## Funding

This study was financially supported by the National Natural Science Foundation of China (81870938 and 82071303), the Natural Science Foundation of Shandong province (ZR2019ZD32 and ZR2020QH107), the Youth Innovation Team of Shandong Universities (2019KJK001), and the Taishan Scholar Project, Academic Promotion Program of Shandong First Medical University (2019LJ001 and 2019QL016).

## Conflict of Interest

The author(s) declared that this work was conducted in the absence of any commercial or financial relationships that could be construed as a potential conflict of interest.

The authors S-xL, H-xL, HY, M-fY, H-jY, Z-yZ, and B-lS shared an affiliation with the editor Yuzhen Xu.

## Publisher's Note

All claims expressed in this article are solely those of the authors and do not necessarily represent those of their affiliated organizations, or those of the publisher, the editors and the reviewers. Any product that may be evaluated in this article, or claim that may be made by its manufacturer, is not guaranteed or endorsed by the publisher.

## Abbreviations

EBI: early brain injury
SAH: subarachnoid hemorrhage
HSP27: Heat shock protein 27
aSAH: aneurysmal subarachnoid hemorrhage
CSF: cerebrospinal fluid
HH: Hunt and Hess
WFNS: World Federation of Neurological Surgeons
MKK4: mitogen-activated protein kinase kinase 4
JNK: c-Jun N-terminal kinase
TAT: transactivating regulatory protein
NPH: normal pressure hydrocephalus
ELISA: enzyme linked immunosorbent assay
AAV: adeno-associated virus
TUNEL: terminal deoxynucleotidyl transferase-mediated dUTP nick end labeling.

## References

[B1] Akbar M. T. Lundberg A. M. Liu K. Vidyadaran S. Wells K. E. Dolatshad H.. (2003). The neuroprotective effects of heat shock protein 27 overexpression in transgenic animals against kainate-induced seizures and hippocampal cell death. J. Biol. Chem. 278, 19956–19965. 10.1074/jbc.M207073200 12639970

[B2] An J. J. Lee Y. P. Kim S. Y. Lee S. H. Lee M. J. Jeong M. S.. (2008). Transduced human PEP-1-heat shock protein 27 efficiently protects against brain ischemic insult. FEBS J. 275, 1296–1308. 10.1111/j.1742-4658.2008.06291.x 18279381

[B3] Bruey J. M. Ducasse C. Bonniaud P. Ravagnan L. Susin S. A. Diaz-Latoud C.. (2000). Hsp27 negatively regulates cell death by interacting with cytochrome c. Nat. Cell Biol. 2, 645–652. 10.1038/35023595 10980706

[B4] Cao G. Pei W. Ge H. Liang Q. Luo Y. Sharp F. R.. (2002). In Vivo delivery of a Bcl-xL fusion protein containing the TAT protein transduction domain protects against ischemic brain injury and neuronal apoptosis. J. Neurosci. 22, 5423–5431. 10.1523/JNEUROSCI.22-13-05423.2002 12097494 PMC6758230

[B5] Chen S. Feng H. Sherchan P. Klebe D. Zhao G. Sun X.. (2014). Controversies and evolving new mechanisms in subarachnoid hemorrhage. Prog. Neurobiol. 115, 64–91. 10.1016/j.pneurobio.2013.09.002 24076160 PMC3961493

[B6] Dong Y. Guo Z. N. Li Q. Ni W. Gu H. Gu Y. X.. (2019). Chinese Stroke Association guidelines for clinical management of cerebrovascular disorders: executive summary and 2019 update of clinical management of spontaneous subarachnoid haemorrhage. Stroke Vasc. Neurol. 4, 176–181. 10.1136/svn-2019-000296 32030200 PMC6979866

[B7] Duan W. Pan Y. Wang C. Wang Y. Zhao X. Wang Y.. (2018). Risk factors and clinical impact of delayed cerebral ischemia after aneurysmal subarachnoid hemorrhage: analysis from the china national stroke registry. Neuroepidemiology. 50, 128–136. 10.1159/000487325 29529609

[B8] Fujii M. Yan J. Rolland W. B. Soejima Y. Caner B. Zhang J. H. (2013). Early brain injury, an evolving frontier in subarachnoid hemorrhage research. Transl. Stroke Res. 4, 432–446. 10.1007/s12975-013-0257-2 23894255 PMC3719879

[B9] Garrido C. Bruey J. M. Fromentin A. Hammann A. Arrigo A. P. Solary E. (1999). HSP27 inhibits cytochrome c-dependent activation of procaspase-9. FASEB J. 13, 2061–2070. 10.1096/fasebj.13.14.2061 10544189

[B10] Helbok R. Schiefecker A. J. Beer R. Dietmann A. Antunes A. P. Sohm F.. (2015). Early brain injury after aneurysmal subarachnoid hemorrhage: a multimodal neuromonitoring study. Crit. Care. 19, 75. 10.1186/s13054-015-0809-9 25887441 PMC4384312

[B11] Iliff J. J. Lee H. Yu M. Feng T. Logan J. Nedergaard M.. (2013). Brain-wide pathway for waste clearance captured by contrast-enhanced MRI. J. Clin. Invest. 123, 1299–1309. 10.1172/JCI67677 23434588 PMC3582150

[B12] Kooijman E. Nijboer C. H. Van Velthoven C. T. Kavelaars A. Kesecioglu J. Heijnen C. J. (2014). The rodent endovascular puncture model of subarachnoid hemorrhage: mechanisms of brain damage and therapeutic strategies. J. Neuroinflam. 11, 2. 10.1186/1742-2094-11-2 24386932 PMC3892045

[B13] Kostenko S. Moens U. (2009). Heat shock protein 27 phosphorylation: kinases, phosphatases, functions and pathology. Cell Mol. Life Sci. 66, 3289–3307. 10.1007/s00018-009-0086-3 19593530 PMC11115724

[B14] Kwan K. Arapi O. Wagner K. E. Schneider J. Sy H. L. Ward M. F.. (2019). Cerebrospinal fluid macrophage migration inhibitory factor: a potential predictor of cerebral vasospasm and clinical outcome after aneurysmal subarachnoid hemorrhage. J. Neurosurg. 1, 1–6. 10.3171/2019.6.JNS19613 31585427

[B15] Leak R. K. Zhang L. Stetler R. A. Weng Z. Li P. Atkins G. B.. (2013). HSP27 protects the blood-brain barrier against ischemia-induced loss of integrity. CNS Neurol. Disord. Drug Target. 12, 325–337. 10.2174/1871527311312030006 23469858 PMC4061290

[B16] Li M. Wang Y. Wang W. Zou C. Wang X. Chen Q. (2017). Recombinant human brain-derived neurotrophic factor prevents neuronal apoptosis in a novel in vitro model of subarachnoid hemorrhage. Neuropsychiatr. Dis. Treat 13, 1013–1021. 10.2147/NDT.S128442 28435271 PMC5388253

[B17] Macomson S. D. Brophy C. M. Miller W. Harris V. A. Shaver E. G. (2002). Heat shock protein expression in cerebral vessels after subarachnoid hemorrhage. Neurosurgery 51, 204–210; discussion 210–201. 10.1097/00006123-200207000-00029 12182419

[B18] Pandey P. Farber R. Nakazawa A. Kumar S. Bharti A. Nalin C.. (2000). Hsp27 functions as a negative regulator of cytochrome c-dependent activation of procaspase-3. Oncogene. 19, 1975–1981. 10.1038/sj.onc.1203531 10803458

[B19] Papa L. Rosenthal K. Silvestri F. Axley J. C. Kelly J. M. Lewis S. B. (2018). Evaluation of alpha-II-spectrin breakdown products as potential biomarkers for early recognition and severity of aneurysmal subarachnoid hemorrhage. Sci. Rep. 8, 13308. 10.1038/s41598-018-31631-y 30190542 PMC6127329

[B20] Park S. Yamaguchi M. Zhou C. Calvert J. W. Tang J. Zhang J. H. (2004). Neurovascular protection reduces early brain injury after subarachnoid hemorrhage. Stroke. 35, 2412–2417. 10.1161/01.STR.0000141162.29864.e9 15322302

[B21] Prunell G. F. Mathiesen T. Diemer N. H. Svendgaard N. A. (2003). Experimental subarachnoid hemorrhage: subarachnoid blood volume, mortality rate, neuronal death, cerebral blood flow, and perfusion pressure in three different rat models. Neurosurgery 52, 165–175; discussion 175–166. 10.1097/00006123-200301000-00022 12493115

[B22] Sabri M. Lass E. Macdonald R. L. (2013). Early brain injury: a common mechanism in subarachnoid hemorrhage and global cerebral ischemia. Stroke Res. Treat. 2013, 394036. 10.1155/2013/394036 23533958 PMC3603523

[B23] Satoh M. Tang J. Nanda A. Zhang J. H. (2003). Heat shock proteins expression in brain stem after subarachnoid hemorrhage in rats. Acta Neurochir. Suppl. 86, 477–482. 10.1007/978-3-7091-0651-8_98 14753490

[B24] Sehba F. A. Hou J. Pluta R. M. Zhang J. H. (2012). The importance of early brain injury after subarachnoid hemorrhage. Prog Neurobiol. 97, 14–37. 10.1016/j.pneurobio.2012.02.003 22414893 PMC3327829

[B25] Shan Q. Ma F. Wei J. Li H. Ma H. Sun P. (2020). Physiological functions of heat shock proteins. Curr. Protein Pept. Sci. 21, 751–760. 10.2174/1389203720666191111113726 31713482

[B26] Shan R. Liu N. Yan Y. Liu B. (2021). Apoptosis, autophagy and atherosclerosis: relationships and the role of Hsp27. Pharmacol. Res. 166, 105169. 10.1016/j.phrs.2020.105169 33053445

[B27] Sharp F. R. Zhan X. Liu D. Z. (2013). Heat shock proteins in the brain: role of Hsp70, Hsp 27, and HO-1 (Hsp32) and their therapeutic potential. Transl. Stroke Res. 4, 685–692. 10.1007/s12975-013-0271-4 24323422 PMC3858824

[B28] Shi Y. Jiang X. Zhang L. Pu H. Hu X. Zhang W.. (2017). Endothelium-targeted overexpression of heat shock protein 27 ameliorates blood-brain barrier disruption after ischemic brain injury. Proc. Natl. Acad Sci. U S A. 114, E1243–E1252. 10.1073/pnas.1621174114 28137866 PMC5320958

[B29] Stetler R. A. Cao G. Gao Y. Zhang F. Wang S. Weng Z.. (2008). Hsp27 protects against ischemic brain injury via attenuation of a novel stress-response cascade upstream of mitochondrial cell death signaling. J. Neurosci. 28, 13038–13055. 10.1523/JNEUROSCI.4407-08.2008 19052195 PMC2614130

[B30] Stetler R. A. Gao Y. Signore A. P. Cao G. Chen J. (2009). HSP27: mechanisms of cellular protection against neuronal injury. Curr. Mol. Med. 9, 863–872. 10.2174/156652409789105561 19860665 PMC2775412

[B31] Stetler R. A. Gao Y. Zhang L. Weng Z. Zhang F. Hu X.. (2012). Phosphorylation of HSP27 by protein kinase D is essential for mediating neuroprotection against ischemic neuronal injury. J. Neurosci. 32, 2667–2682. 10.1523/JNEUROSCI.5169-11.2012 22357851 PMC3403749

[B32] Toth M. E. Szegedi V. Varga E. Juhasz G. Horvath J. Borbely E.. (2013). Overexpression of Hsp27 ameliorates symptoms of Alzheimer's disease in APP/PS1 mice. Cell Stress Chaper. 18, 759–771. 10.1007/s12192-013-0428-9 23605646 PMC3789881

[B33] Unterberg A. W. Sakowitz O. W. Sarrafzadeh A. S. Benndorf G. Lanksch W. R. (2001). Role of bedside microdialysis in the diagnosis of cerebral vasospasm following aneurysmal subarachnoid hemorrhage. J. Neurosurg. 94, 740–749. 10.3171/jns.2001.94.5.0740 11354405

[B34] Vendredy L. Adriaenssens E. Timmerman V. (2020). Small heat shock proteins in neurodegenerative diseases. Cell Stress Chaperones 25, 679–699. 10.1007/s12192-020-01101-4 32323160 PMC7332613

[B35] Voss O. H. Batra S. Kolattukudy S. J. Gonzalez-Mejia M. E. Smith J. B. Doseff A. I. (2007). Binding of caspase-3 prodomain to heat shock protein 27 regulates monocyte apoptosis by inhibiting caspase-3 proteolytic activation. J. Biol. Chem. 282, 25088–25099. 10.1074/jbc.M701740200 17597071

[B36] Wang W. Han P. Xie R. Yang M. Zhang C. Mi Q.. (2019). TAT-mGluR1 attenuation of neuronal apoptosis through prevention of MGluR1alpha truncation after experimental subarachnoid hemorrhage. ACS Chem. Neurosci. 10, 746–756. 10.1021/acschemneuro.8b00531 30339347

[B37] Wasik N. Sokol B. Holysz M. Manko W. Juszkat R. Jagodzinski P. P.. (2017). Clusterin, a new cerebrospinal fluid biomarker in severe subarachnoid hemorrhage: a pilot study. World Neurosurg. 107, 424–428. 10.1016/j.wneu.2017.08.006 28803177

[B38] Wu Q. Qi L. Li H. Mao L. Yang M. Xie R.. (2017). Roflumilast reduces cerebral inflammation in a rat model of experimental subarachnoid hemorrhage. Inflammation 40, 1245–1253. 10.1007/s10753-017-0567-8 28451841 PMC6193485

[B39] Xie L. Kang H. Xu Q. Chen M. J. Liao Y. Thiyagarajan M.. (2013). Sleep drives metabolite clearance from the adult brain. Science. 342, 373–377. 10.1126/science.1241224 24136970 PMC3880190

[B40] Yang Y. Chen S. Zhang J. M. (2017). The updated role of oxidative stress in subarachnoid hemorrhage. Curr. Drug. Deliv. 14, 832–842. 10.2174/1567201813666161025115531 27784210

[B41] Zhang C. Jiang M. Wang W. Q. Zhao S. J. Yin Y. X. Mi Q. J.. (2020). Selective mGluR1 negative allosteric modulator reduces blood-brain barrier permeability and cerebral edema after experimental subarachnoid hemorrhage. Transl. Stroke Res. 11, 799–811. 10.1007/s12975-019-00758-z 31833035

[B42] Zhang Z. Liu J. Fan C. Mao L. Xie R. Wang S.. (2018). The GluN1/GluN2B NMDA receptor and metabotropic glutamate receptor 1 negative allosteric modulator has enhanced neuroprotection in a rat subarachnoid hemorrhage model. Exp. Neurol. 301, 13–25. 10.1016/j.expneurol.2017.12.005 29258835

[B43] Zhang Z. Y. Sun B. L. Yang M. F. Li D. W. Fang J. Zhang S. (2015). Carnosine attenuates early brain injury through its antioxidative and anti-apoptotic effects in a rat experimental subarachnoid hemorrhage model. Cell Mol. Neurobiol. 35, 147–157. 10.1007/s10571-014-0106-1 25179154 PMC11486197

[B44] Zhao C. Ma J. Wang Z. Li H. Shen H. Li X.. (2020). Mfsd2a attenuates blood-brain barrier disruption after sub-arachnoid hemorrhage by inhibiting caveolae-mediated transcellular transport in rats. Transl. Stroke Res. 11, 1012–1027. 10.1007/s12975-019-00775-y 31907728

[B45] Zhou M. L. Shi J. X. Hang C. H. Cheng H. L. Qi X. P. Mao L.. (2007). Potential contribution of nuclear factor-kappaB to cerebral vasospasm after experimental subarachnoid hemorrhage in rabbits. J. Cereb. Blood Flow Metab. 27, 1583–1592. 10.1038/sj.jcbfm.9600456 17293842

