# Corrigendum: TAT-HSP27 peptide improves neurologic deficits via reducing apoptosis after experimental subarachnoid hemorrhage

**DOI:** 10.3389/fncel.2024.1414704

**Published:** 2024-06-05

**Authors:** Xiao-yan Zhou, Jing-yi Sun, Wei-qi Wang, Shu-xian Li, Han-xia Li, Hui-juan Yang, Ming-feng Yang, Hui Yuan, Zong-yong Zhang, Bao-liang Sun, Jin-Xiang Han

**Affiliations:** ^1^Department of Biochemistry and Molecular Biology, School of Basic Medical Sciences, Shandong University, Ji'nan, China; ^2^Department of Neurosurgery, First Affiliated Hospital of Shandong First Medical University and Shandong Academy of Medical Sciences, Ji'nan, China; ^3^Biomedical Sciences College and Shandong Medicinal Biotechnology Centre, Shandong First Medical University and Shandong Academy of Medical Sciences, Ji'nan, China; ^4^Key Lab for Biotech-Drugs of National Health Commission, Shandong First Medical University and Shandong Academy of Medical Sciences, Ji'nan, China; ^5^Department of Orthopedics, Shandong Provincial Hospital Affiliated to Shandong First Medical University and Shandong Academy of Medical Sciences, Jinan, China; ^6^Department of Neurology, Shandong Provincial Hospital Affiliated to Shandong First Medical University and Shandong Academy of Medical Sciences, Jinan, China; ^7^Department of Neurology, Key Laboratory of Cerebral Microcirculation, Second Affiliated Hospital of Shandong First Medical University and Shandong Academy of Medical Sciences, Taian, China

**Keywords:** subarachnoid hemorrhage, HSP27, cell apoptosis, neurologic deficits, TAT-HSP27_65 − 90_ peptide

In the published article, there was an error in the images of Figure 7D and Figure 8A as published, where in Figure 7D the incorrect phase contrast image of 31-60 group was used and in Figure 8A the brain picture of vehicle group was incorrect.

The corrected Figure 7D and Figure 8A appear in Figure 7 and Figure 8 below.

**Figure 7:**
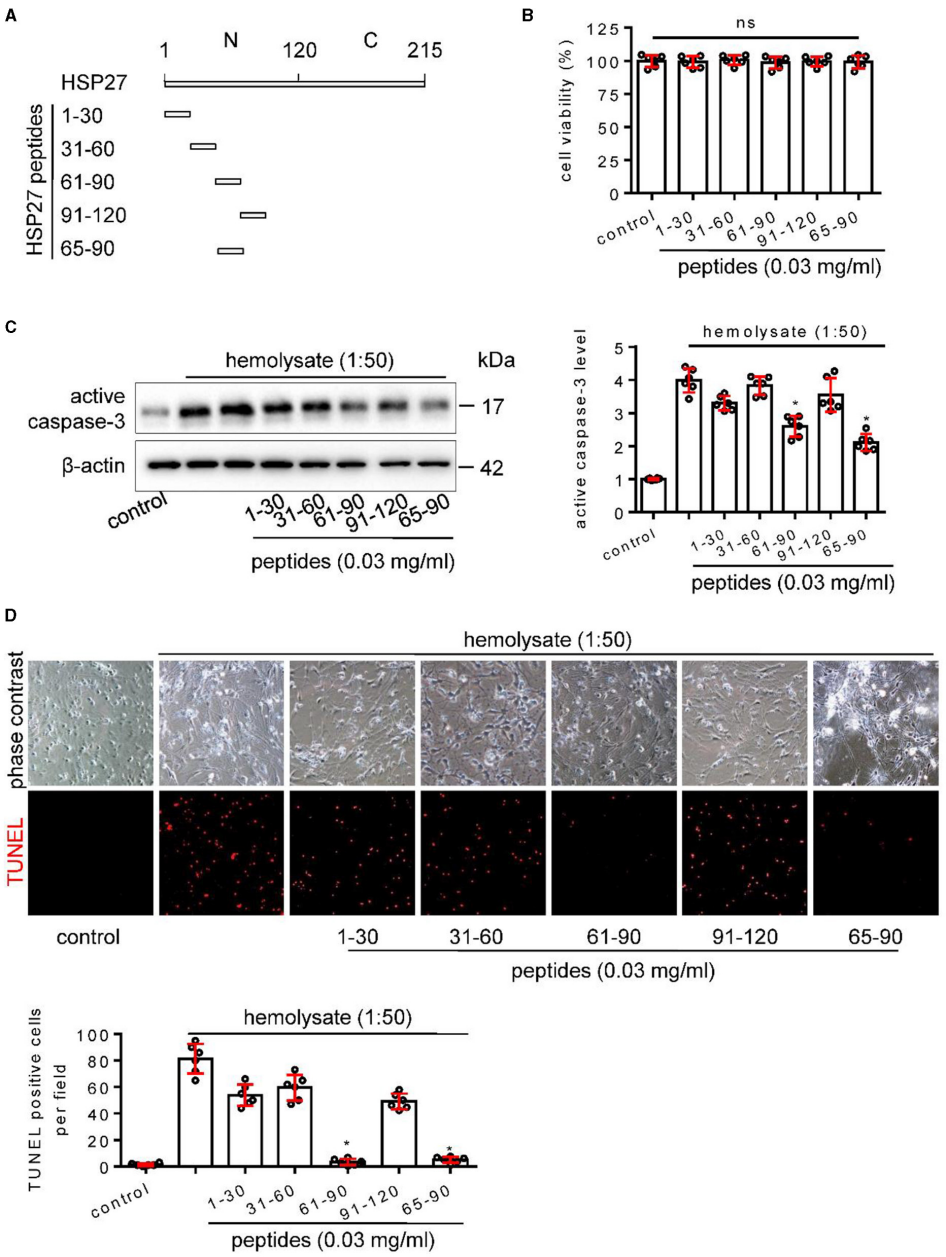
Effect of HSP27 peptides on hemolysate-induced cell apoptosis in primary cortical neurons. **(A)** Schematic representation of various HSP27 peptides. **(B)** Hsp27 peptides have no effect on cell viability in primary cortical neurons; Cortical neurons were treated with indicated HSP27 peptides (0.03 mg/ml) in medium (1:50) for 24 h. Cell viability was measured with Cell Counting Kit-8 (CCK-8) and normalized to control. **(C, D)** Cortical neurons were treated with hemolysate in medium (1:50) or plus indicated HSP27 peptides (0.03 mg/ml) for 24 h. **(C)** Active caspase-3 levels in each group were detected by Western blot, β-actin serves as a control, and quantification of optical density was normalized to control. **(D)** Representative images of cortical neurons (phase contrast, ×200) and TUNEL staining (red, ×200), and quantification of TUNEL-positive cells from each group was performed. Data are mean ± SD, *n* = 6, ^*^p < 0.05 vs. hemolysate treatment, ANOVA with Bonferroni's multiple comparisons test.

**Figure 8:**
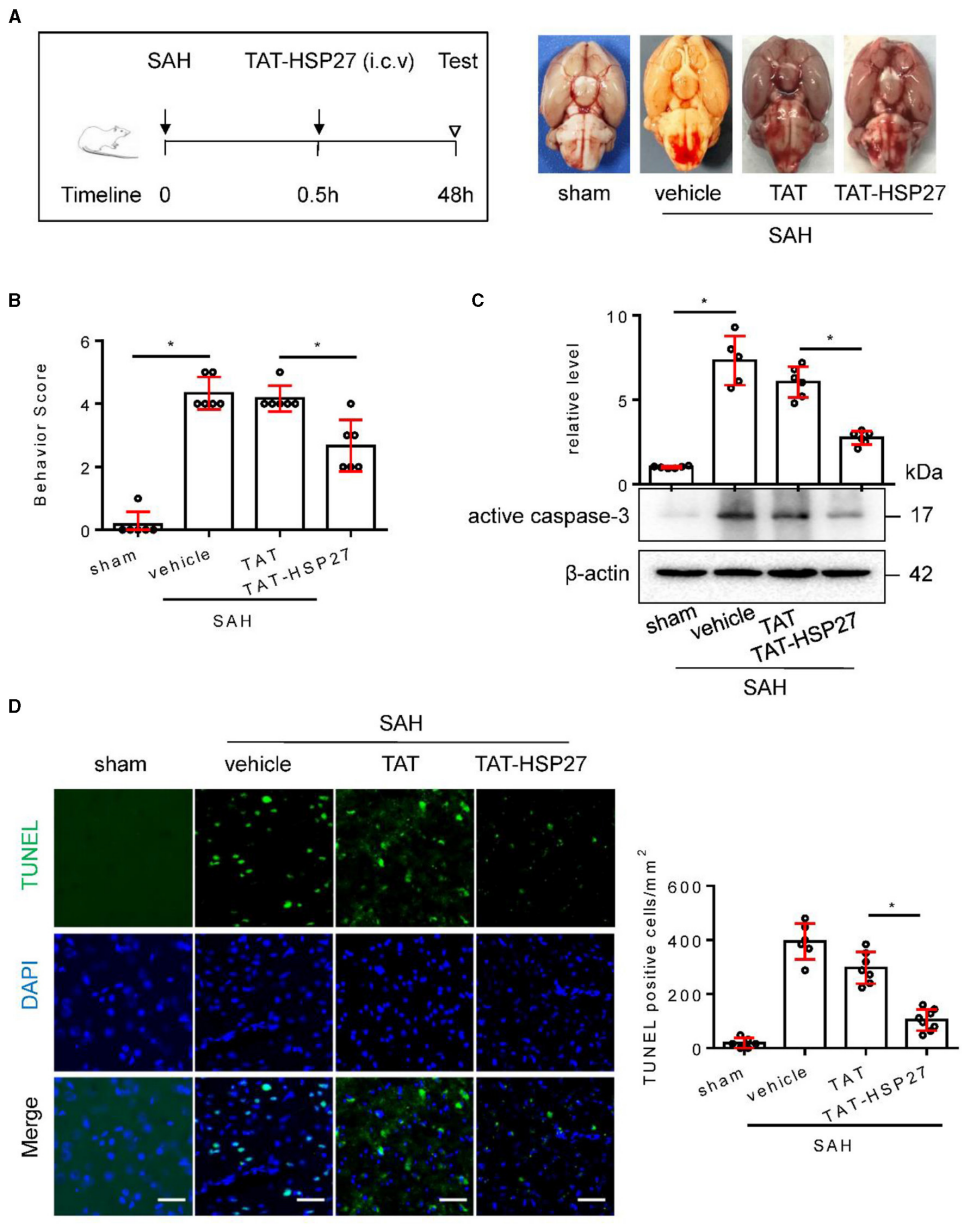
TAT- HSP27_65 − 90_ peptide attenuated neurological deficits and cell apoptosis after SAH in rats. **(A)** Experimental design; representative images of rat brain on day 2 after surgery for the sham, SAH + vehicle, SAH + TAT peptide (SAH + TAT), and SAH + TAT-HSP27_65 − 90_ peptide (SAH + TAT-HSP27) groups. **(B)** Behavior scores of each group were assessed at 48 h, *n* = 6. **(C)** The basal cortex was collected on day 2 following SAH from the sham (*n* = 6), SAH + vehicle (*n* = 5), SAH + TAT (*n* = 6), and SAH + TAT-HSP27 (*n* = 6) groups; homogenates were blotted with anti-active caspase-3 and anti-β-actin, and quantification of optical density was normalized to sham group. **(D)** Coronal sections from the sham (*n* = 6), SAH + vehicle (*n* = 6), SAH + TAT (*n* = 7), and SAH + TAT-HSP27 (*n* = 8) group reperfusion on day 2 after SAH, subjected to immunostaining for the TUNEL (green) in the basal cortex. Quantification was performed by counting the TUNEL positive cells per mm^2^ region in the basal cortex, scale bar = 50 μm. Data are mean ± SD, ^*^p < 0.05 vs. hemolysate treatment, ANOVA with Bonferroni's multiple comparisons test.

The authors apologize for these errors and state that they do not change the scientific conclusions of the article in any way. The original article has been updated.

