# Correction: TAT-HSP27 peptide improves neurologic deficits and reduces apoptosis after experimental subarachnoid hemorrhage

**DOI:** 10.3389/fncel.2026.1911280

**Published:** 2026-07-21

**Authors:** Xiao-yan Zhou, Jing-yi Sun, Wei-qi Wang, Shu-xian Li, Han-xia Li, Hui-juan Yang, Ming-feng Yang, Hui Yuan, Zong-yong Zhang, Bao-liang Sun, Jin-Xiang Han

**Affiliations:** 1 Department of Biochemistry and Molecular Biology, School of Basic Medical Sciences, Shandong University, Ji'nan, China; 2 Department of Neurosurgery, First Affiliated Hospital of Shandong First Medical University and Shandong Academy of Medical Sciences, Ji'nan, China; 3 Biomedical Sciences College and Shandong Medicinal Biotechnology Centre, Shandong First Medical University and Shandong Academy of Medical Sciences, Ji'nan, China; 4 Key Lab for Biotech-Drugs of National Health Commission, Shandong First Medical University and Shandong Academy of Medical Sciences, Ji'nan, China; 5 Department of Orthopedics, Shandong Provincial Hospital Affiliated to Shandong First Medical University and Shandong Academy of Medical Sciences, Jinan, China; 6 Department of Neurology, Shandong Provincial Hospital Affiliated to Shandong First Medical University and Shandong Academy of Medical Sciences, Jinan, China; 7 Department of Neurology, Key Laboratory of Cerebral Microcirculation, Second Affiliated Hospital of Shandong First Medical University and Shandong Academy of Medical Sciences, Taian, China

**Keywords:** subarachnoid hemorrhage, HSP27, cell apoptosis, neurologic deficits, TAT-HSP27_65−90_ peptide

The title of this article was erroneously given as: TAT-HSP27 Peptide improves neurologic deficits via reducing apoptosis after experimental subarachnoid hemorrhage. The correct title of the article is TAT-HSP27 peptide improves neurologic deficits and reduces apoptosis after experimental subarachnoid hemorrhage.

There was a mistake in Table 1 as published. The word “barking” in row 5 of Table 1 in the original version should be deleted. The corrected Table 1 appears below.

**Table 1:** Behavior scores.

Category	Behavior	Score
Appetite	Finished meal	0
	Left meal unfinished	1
	Scarcely ate	2
Activity	Active, walking, or standing	0
	Lying down, walk, and stand with some stimulations	1
	Almost always lying down	2
Deficits	No deficits	0
	Unstable walk	1
	Impossible to walk and stand	2

There was a mistake in Figures 4, 5, 8 as published. The whole brain images in Figures 4A, 5A, 8A have not been taken under the same conditions. The corrected Figures 4, 5, 8 appears below.

**Figure 4:**
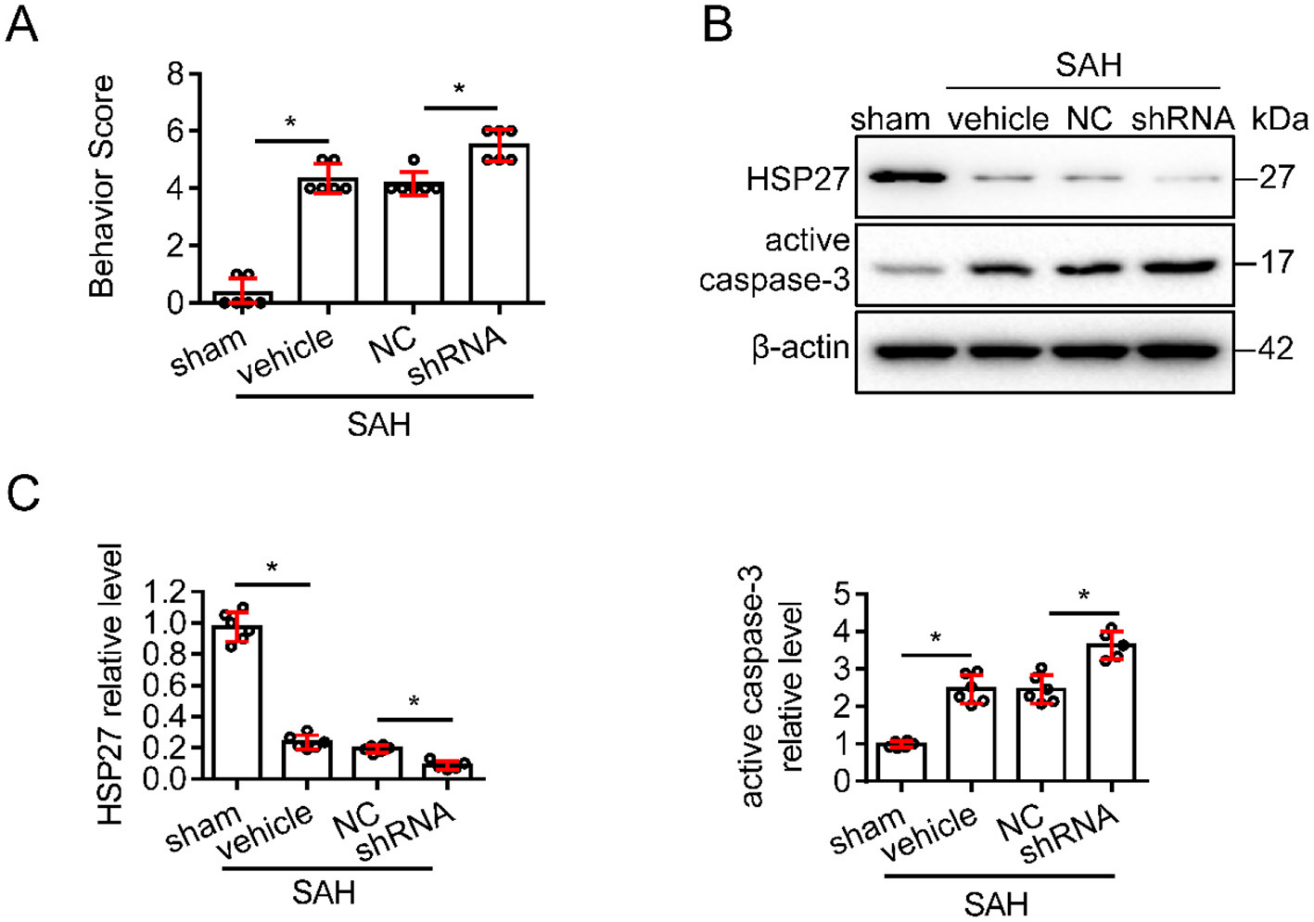
AAV-shRNA targeting HSP27 deteriorated neurological deficit after SAH in rats. **(A)** Representative images of rat brain on day 2 after surgery for the sham, SAH + vehicle, SAH + NC, and SAH + shRNA groups. **(B)** Behavior scores of each group were assessed at 48 h, *n* = 6. **(C)** The basal cortex was collected on day 2 following SAH from the sham (*n* = 6), SAH + vehicle (*n* = 6), SAH + NC (*n* = 6), and SAH + shRNA (*n* = 5) groups; homogenates were blotted with anti-HSP27, anti-active caspase-3, and anti-β-actin, quantification of optical density was normalized to sham group. Data are mean ± SD, ^*^p < 0.05, ANOVA with Bonferroni's multiple comparisons test.

**Figure 5:**
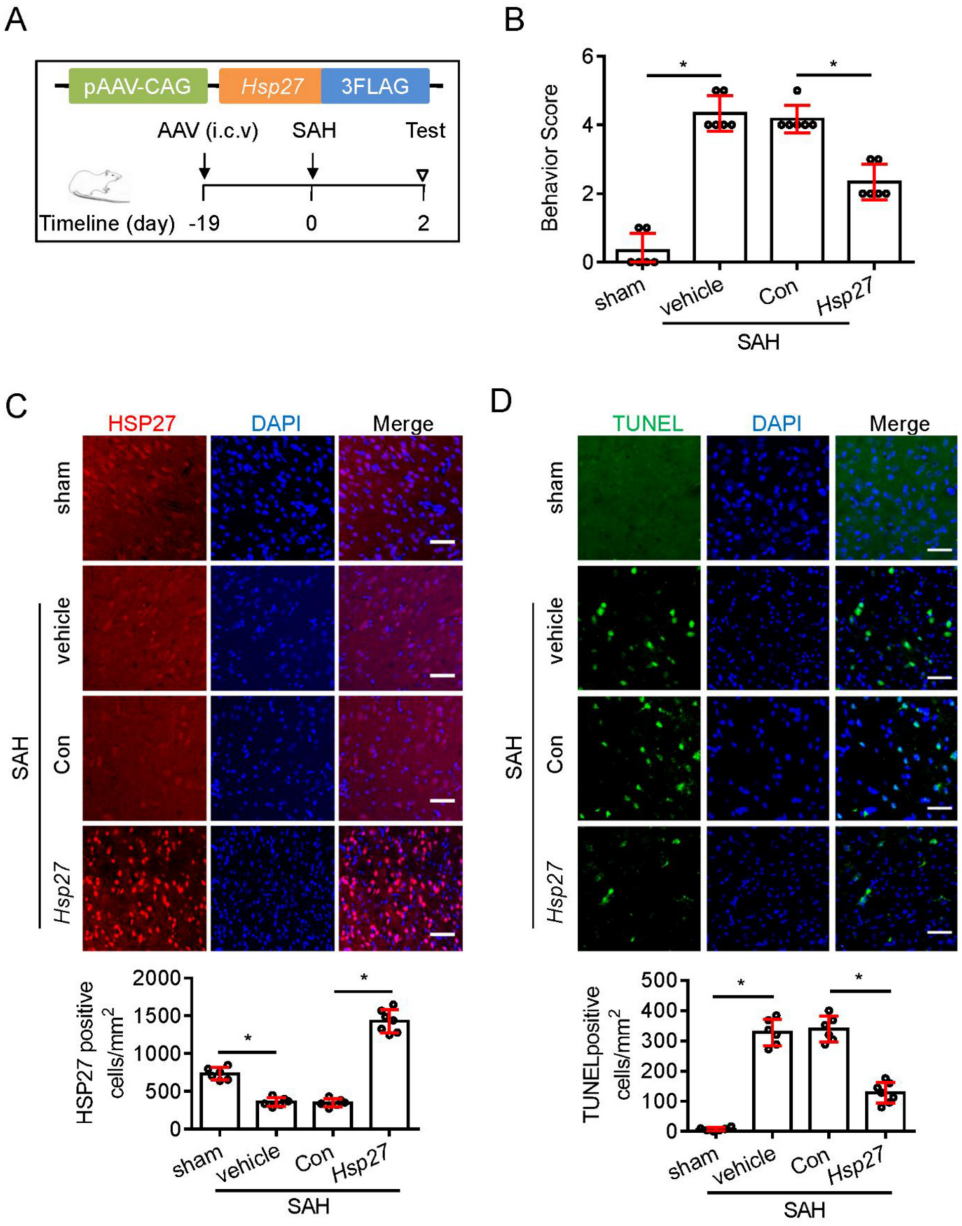
HSP27 overexpression attenuated neurological deficit and cell apoptosis after SAH in rats. **(A)** Schematic of AAV vector encoding HSP27 and experimental design. Representative images of rat brain on day 2 after surgery for the sham, SAH + vehicle, SAH + AAV vector (SAH + Con), and SAH + AAV vector encoding HSP27 (SAH + HSP27) group. **(B)** Behavior scores of each group were assessed at 48 h, *n* = 6. **(C, D)** Coronal sections from the sham (*n* = 6), SAH + vehicle (*n* = 6), SAH + Con (*n* = 6), and SAH + HSP27 (*n* = 7) group reperfusion on day 2 after SAH, subjected to immunostaining for the HSP27 (red) or TUNEL (green) in the basal cortex. Quantification was performed by counting the HSP27 or TUNEL positive cells per mm^2^ region in the basal cortex, scale bar = 50 μm. Data are mean ± SD, ^*^p < 0.05, ANOVA with Bonferroni's multiple comparisons test.

**Figure 8:**
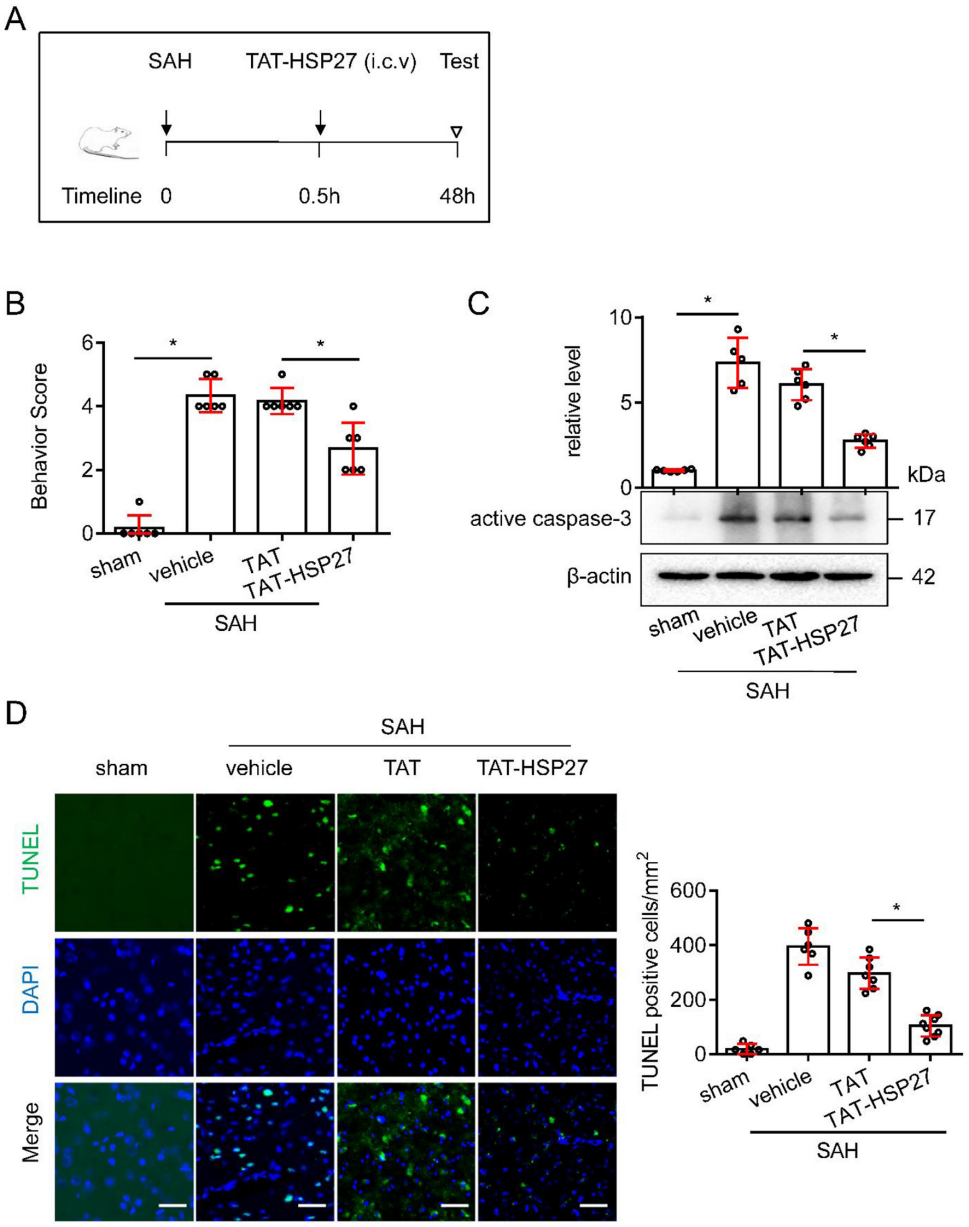
TAT-HSP27_65 − 90_ peptide attenuated neurological deficits and cell apoptosis after SAH in rats. **(A)** Experimental design; representative images of rat brain on day 2 after surgery for the sham, SAH + vehicle, SAH + TAT peptide (SAH + TAT), and SAH + TAT-HSP27_65 − 90_ peptide (SAH + TAT-HSP27) groups. **(B)** Behavior scores of each group were assessed at 48 h, *n* = 6. **(C)** The basal cortex was collected on day 2 following SAH from the sham (*n* = 6), SAH + vehicle (*n* = 5), SAH + TAT (*n* = 6), and SAH + TAT-HSP27 (*n* = 6) groups; homogenates were blotted with anti-active caspase-3 and anti-β-actin, and quantification of optical density was normalized to sham group. **(D)** Coronal sections from the sham (*n* = 6), SAH + vehicle (*n* = 6), SAH + TAT (*n* = 7), and SAH + TAT-HSP27 (*n* = 8) group reperfusion on day 2 after SAH, subjected to immunostaining for the TUNEL (green) in the basal cortex. Quantification was performed by counting the TUNEL positive cells per mm^2^ region in the basal cortex, scale bar = 50 μm. Data are mean ± SD, ^*^p < 0.05 vs. hemolysate treatment, ANOVA with Bonferroni's multiple comparisons test.

In the abstract, “… and exerts protective role on cells,” the indefinite article is missing. Hsp27 does not exert a “role.” “In rat SAH model…,” the definite article is missing. “and then obviously declined…,” “obviously” is the wrong expression. TAT is not the abbreviation for “trans-activating regulatory protein.” The gene *Hsp27* is not italicized. This has been corrected to read:

Cell apoptosis plays an important role in early brain injury (EBI) after subarachnoid hemorrhage (SAH). Heat shock protein 27 (HSP27), a member of the small heat shock protein family, is induced by various stress factors, and has a protective effect on cells. However, the role of HSP27 in brain injury after SAH needs to be further clarified. Here, we report that HSP27 level of cerebrospinal fluid (CSF) is clearly increased at day 1 in patients with aneurysmal SAH (aSAH). That increase is related to the clinical severity of the damage, as assessed by the grade of Hunt and Hess (HH), World Federation of Neurological Surgeons (WFNS) and Fisher. In the rat SAH model, HSP27 of CSF is increased at first and then declined; overexpression of *Hsp27*, not knockdown of *Hsp27*, attenuates SAH-induced neurological deficit and cell apoptosis in basal cortex; overexpression of *Hsp27* effectively suppresses SAH-elevated the activation of Mitogen-Activated Protein Kinase Kinase 4 (MKK4), the c-Jun N-terminal kinase (JNK), c-Jun and caspase-3. In an *in vitro* hemolysate-damaged cortical neuron model, HSP27_65 − 90_ peptide effectively inhibits hemolysate-induced neuron death. Furthermore, TAT-HSP27_65 − 90_ peptide, a fusion peptide consisting of Trans-Activator of Transcription (TAT) of HIV and HSP27_65 − 90_ peptide, effectively attenuates SAH-induced neurological deficit and cell apoptosis in basal cortex in rats. Altogether, our results suggest TAT-HSP27 peptide improves neurologic deficits and reduces apoptosis.

A correction has been made to the Section, in the third line of the first paragraph of the **Introduction**, “aneurysm” should be “aneurysms.” The gene *Hsp27* should be italicized.

The conflict of interest statement was erroneously given as “the authors declare that they have no conflict of interest”. The correct conflict of interest statement is:

The original version of this article has been updated.

