# Supplementary figures and images for: TAT-HSP27 Peptide Improves Neurologic Deficits and Reduces Apoptosis After Experimental Subarachnoid Hemorrhage
Source: Front Cell Neurosci. 2022 Apr 28;16:878673. doi: 10.3389/fncel.2022.878673 (PMC9096089; doi:10.3389/fncel.2022.878673)

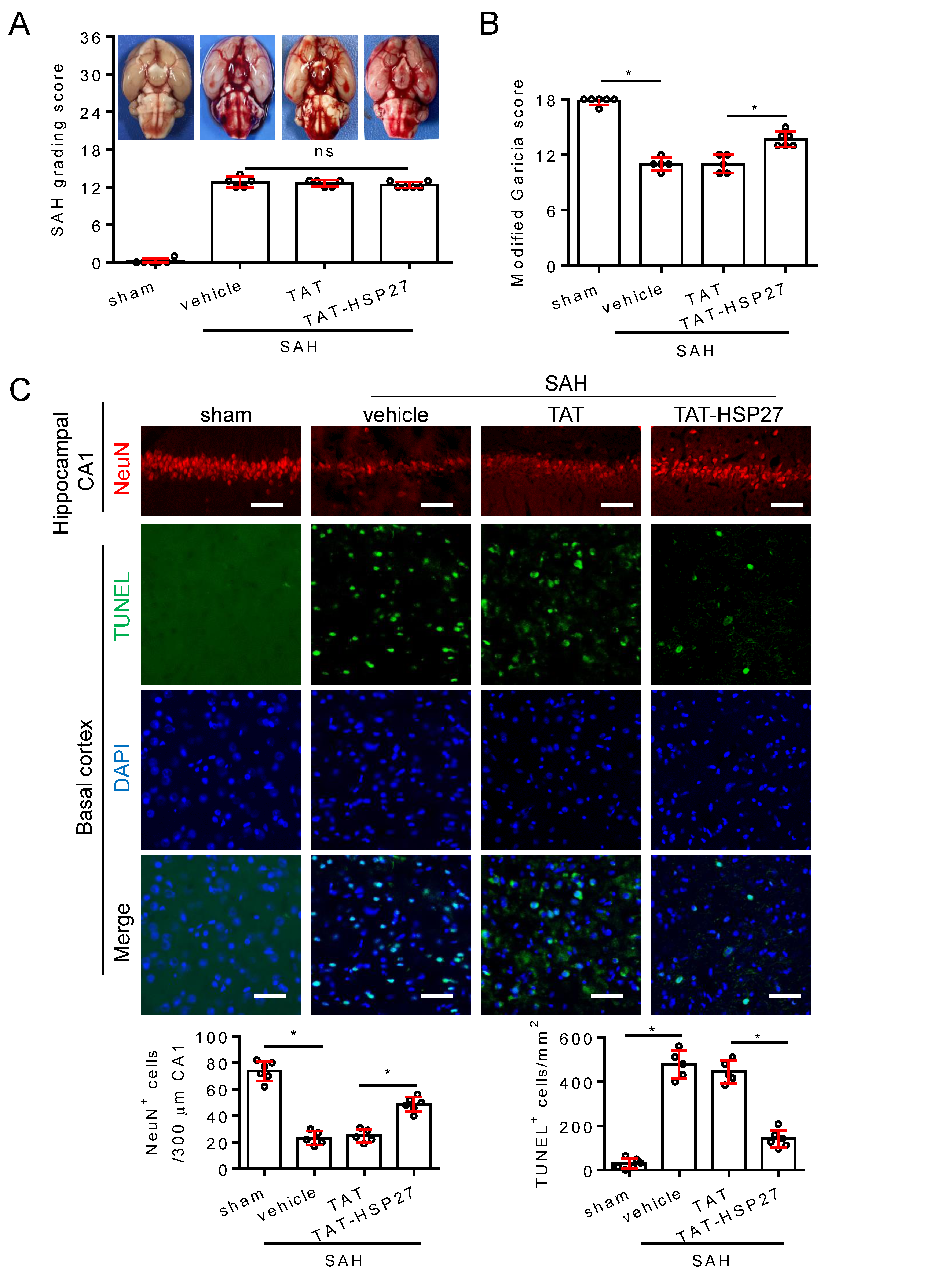

Supplement: Supplementary file 1 [file Image_1.TIFF]
